# Genetic Variability in Balkan Paleoendemic Resurrection Plants *Ramonda serbica* and *R. nathaliae* Across Their Range and in the Zone of Sympatry

**DOI:** 10.3389/fpls.2022.873471

**Published:** 2022-04-28

**Authors:** Maja Lazarević, Sonja Siljak-Yakovlev, Agathe Sanino, Marjan Niketić, Françoise Lamy, Damien D. Hinsinger, Gordana Tomović, Branka Stevanović, Vladimir Stevanović, Thierry Robert

**Affiliations:** ^1^Department of Plant Ecology and Phytogeography, Faculty of Biology, University of Belgrade, Belgrade, Serbia; ^2^Ecologie Systématique Evolution, CNRS, AgroParisTech, Univ. Paris-Sud, Université Paris-Saclay, Gif-sur-Yvette, France; ^3^Natural History Museum, Belgrade, Serbia; ^4^Serbian Academy of Sciences and Arts, Belgrade, Serbia; ^5^Department of Biology, University of Versailles-Saint-Quentin, Versailles, France; ^6^Département Biologie et Amélioration des Plantes, Polymorphisme des Génomes Végétaux, INRAE, Evry, France; ^7^Biology Department, Sorbonne Université, Paris, France

**Keywords:** AFLP, gene flow, genetic diversity, genome size, Gesneriaceae, interploidy hybridization, mixed-ploidy zones, sympatry

## Abstract

The genus *Ramonda* includes three Paleoendemic and Tertiary relict species that survived in refugial habitats of the Balkan Peninsula (*R. nathaliae* and *R. serbica*) and the Iberian Peninsula (*R. myconi*). They are all “resurrection plants,” a rare phenomenon among flowering plants in Europe. *Ramonda myconi* and *R. nathaliae* are diploids (2*n* = 2*x* = 48), while *R. serbica* is a hexaploid (2*n* = 6*x* = 144). The two Balkan species occur in sympatry in only two localities in eastern Serbia, where tetraploid potential hybrids (2*n* = 4*x* = 96) were found. This observation raised questions about the existence of gene flow between the two species and, more generally, about the evolutionary processes shaping their genetic diversity. To address this question, genetic markers (AFLP) and an estimate of genome size variation were used in a much larger sample and at a larger geographic scale than previously. The combination of AFLP markers and genome size results suggested ongoing processes of interspecific and interploidy hybridization in the two sites of sympatry. The data also showed that interspecific gene flow was strictly confined to sympatry. Elsewhere, both *Ramonda* species were characterized by low genetic diversity within populations and high population differentiation. This is consistent with the fact that the two species are highly fragmented into small and isolated populations, likely a consequence of their postglacial history. Within sympatry, enormous variability in cytotypes was observed, exceeding most reported cases of mixed ploidy in complex plant species (from 2*x* to >8*x*). The AFLP profiles of non-canonical ploidy levels indicated a diversity of origin pathways and that backcrosses probably occur between tetraploid interspecific hybrids and parental species. The question arises whether this diversity of cytotypes corresponds to a transient situation. If not, the question arises as to the genetic and ecological mechanisms that allow this diversity to be maintained over time.

## Introduction

The Mediterranean basin is one of the richest regions in terms of biodiversity, with 23,000 to 25,000 plant species (Thompson, 2020). The Balkan Peninsula, as part of the Mediterranean Basin, one of the global hotspots of biodiversity (Myers et al., 2000), is one of the centers of species richness and endemism in Europe with more than 8,000 plant species, of which about 30% are endemic (Turill, 1929; Stevanović et al., 2007). Several geological, climatic, and biogeographical factors have been identified that have contributed to the richness and endemism of the flora of this region (Hewitt, 1996, 1999; Stevanović et al., 1999, 2007; Krstić et al., 2012; Kougioumoutzis et al., 2021). Polyploidization and interspecific hybridization have played an important role especially in the evolution of many plant taxa in the Balkan Peninsula, e.g., *Alyssum* (Španiel et al., 2017), *Cardamine* (Šlenker et al., 2021), *Centaurium* (Banjanac et al., 2017), *Cerastium* (Niketić et al., 2013, 2021), *Cotoneaster* (Bogunić et al., 2021), *Edraianthus* (Lakušić et al., 2009), *Knautia* (Frajman et al., 2015), *Luzula* (Bačič et al., 2016), *Sesleria* (Kuzmanović et al., 2013; Lazarević et al., 2015), *Sorbus* (Hajrudinović et al., 2015), *Veronica* (Padilla-García et al., 2018).

Polyploidization and hybridization are considered important forces in plant evolution (Wendel, 2000; Mallet, 2007; Soltis et al., 2009; Jiao et al., 2011; Van de Peer et al., 2021). Polyploids may differ from their diploid relatives in their morphology, phenology, physiology, biotic interactions, distribution, preferences for ecological niches, and invasiveness (e.g., Levin, 2002; Raabová et al., 2008; Maherali et al., 2009; te Beest et al., 2012; Weiss-Schneeweiss et al., 2013; Stutz et al., 2016; Decanter et al., 2020; Ulum et al., 2021). Many studies suggest better fitness of polyploids (Comai, 2005; Maherali et al., 2009; te Beest et al., 2012; Rey et al., 2017; Van de Peer et al., 2021), but opposite results can also be found in the literature (Nunvářová Kabátová et al., 2019). Polyploid individuals may be favored under stressful conditions, including major climatic or geological changes, and may provide an advantage in surviving and adapting to such conditions (Otto and Whitton, 2000; Comai, 2005; Weiss-Schneeweiss et al., 2013; Van de Peer et al., 2017, 2021; Decanter et al., 2020). Climate change may also lead to shifts in species distributions that bring previously separate species into contact and allow hybridization, including related species that have evolved to different ploidy levels (Vallejo-Marín and Hiscock, 2016).

There is increasing interest in the genus *Ramonda* as a model to study the role of interploidy gene flow in enhancing genetic and phenotypic diversity and the ecological factors that may contribute to the maintenance of cytotype diversity. The genus *Ramonda* Rich. (Gesneriaceae) includes three species—*R. myconi* (L.) Rchb., *R. nathaliae* Pančić & Petrović and *R. serbica* Pančić. All three species are considered relicts of the Tertiary (Košanin, 1921; Turill, 1929; Petrova et al., 2015), remnants of the tropical-subtropical flora from the time when the climate in southern Europe was warmer and more humid. Apart from being able to survive almost complete desiccation of their aboveground tissues in a state of anabiosis when habitat conditions are dry, and rehydrate when humidity becomes favorable again (Živković et al., 2005; Rakić et al., 2014), the three species also share similar habitats. They occur exclusively on north-facing rocky slopes of gorges, canyons, and mountain ravines, where conditions are wetter and more sheltered, allowing them longer active periods (Košanin, 1921; Rakić et al., 2014). However, *R. myconi* and *R. serbica* are found only on limestone, while *R. nathaliae* also inhabits ultramafic soils (Košanin, 1921; Stevanović and Stevanović, 1985; Lazarević et al., 2013; Rakić et al., 2013). *Ramonda myconi* and *R. nathaliae* are diploids with 2*n* = 2*x* = 48 chromosomes and mean 2C values of 2.59 and 2.32 pg, respectively, while *R. serbica* is hexaploid with 2*n* = 6*x* = 144 and mean 2C = 7.91 pg (Siljak-Yakovlev et al., 2008). Relatively high basic chromosome number (*x* = 24) indicates that the three species may be paleopolyploids (Rakić et al., 2014). Also, based on the similarity of monoploid genome size in *R. myconi* and *R. serbica*, the same authors hypothesized past existence of a common diploid ancestor with 2*n* = 24 chromosomes from which later originated *R. nathaliae* on one side, and *R. myconi* and *R. serbica* on the other.

**Figure 1:**
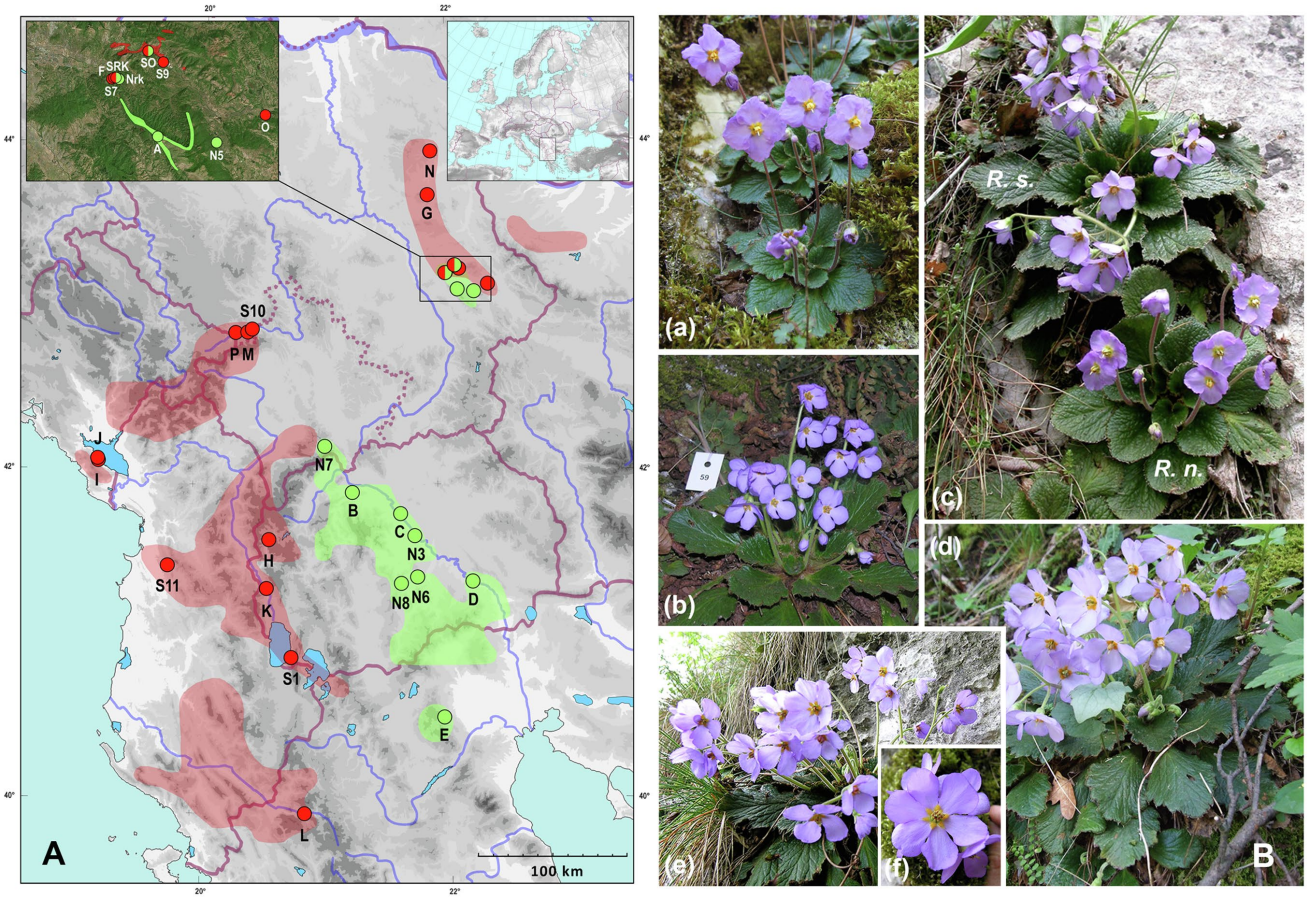
**(A)** Geographic range of *R. nathaliae* and *R. serbica* and distribution map of investigated populations. Green color and green dots represent range and investigated populations of *R. nathaliae*. Red color and red dots represent range and investigated populations of *R. serbica*. Detailed distribution of two species in the zone of sympatry is presented in the upper left inset. Geographical position of the investigated area in Europe is presented in the upper right inset. SO—sympatry at Oblik, SRK—sympatry at Radovanski Kamen. See Table 1 for detailed population legend. **(B)** *R. nathaliae* from population Divljana (a); *R. serbica* from population Radovanski Kamen (b); two species side by side in the sympatry Radovanski Kamen (c); tetraploid hybrid from Radovanski Kamen (d) and hybrid of higher ploidy level [probably backcross; (e)] showing some flowers with six petals (f).

Despite ecological similarities, the ranges of three *Ramonda* species differ, with *R. myconi* inhabiting the NE part of the Iberian Peninsula, while *R. nathaliae* and *R. serbica* are endemics of the Balkan Peninsula. *Ramonda nathaliae* is distributed mainly in the Aegean watershed area (North Macedonia, N Greece and SW Serbia—one locality in SC Kosovo at the foothill of Mt. Šara) with small enclaves in E Serbia (the Black Sea watershed). In contrast, *R. serbica* is mainly present in the areas of the Adriatic watershed (Albania, S and SE Montenegro, W North Macedonia, SW Serbia—including W-SW Kosovo, NW Greece) and to a smaller extent in the Black Sea (SW, E and NE Serbia, NW Bulgaria) and the Aegean watersheds (one population in NW part of North Macedonia; Figure 1A, detailed map in Rakić et al., 2014). The range of the two species in the Balkans overlaps only in E Serbia in a narrow area of sympatry (Figure 1A) discovered only a few decades ago (Stevanović et al., 1986). In only two places in the nearby gorges of the Jelašnica and Nišava rivers (6–7 km apart), *R. nathaliae* and *R. serbica* can be found in the same macrohabitat, in the close proximity to each other opening possibility for hybridization. At the time of discovery of these two sites of sympatry, the authors did not notice any intermediate phenotypes that might indicate hybridization between the two species. However, thanks to a preliminary cytogenetic study at the two sites of sympatry, several individuals with intermediate chromosome number (2*n* = 4*x* = 96) and genome size (2C = 5.14 pg) were found at both locations (Siljak-Yakovlev et al., 2008). The presence of tetraploid plants has suggested the possibility of interploidy and thus interspecific hybridization. While two *Ramonda* species from the Balkan Peninsula can be clearly distinguished on the basis of leaf and flower characteristics (Pančić, 1874, 1884; Petrović, 1882), field and laboratory observations of tetraploid individuals have shown that it is very difficult to distinguish them from the parent species on the basis of morphological traits. Preliminary studies of flower morphology suggest greater similarity of tetraploid hybrids to *R. serbica* (Figure 1B) and very careful use of certain flower characters (the color of the anthers and the angle formed by the lines connecting the petal base and the points of maximum petal width) when attempting to distinguish tetraploid individuals from the canonical representatives of diploid *R. nathaliae* and hexaploid *R. serbica* (Lazarević et al., 2014).

Thus, the genetic status of the tetraploid individuals is unclear. Genetic analyses are needed to advance the study of possible hybridization between these two species. However, cytotype diversity in these two contact zones has not yet been thoroughly evaluated because initial studies were based on only a few individuals (Siljak-Yakovlev et al., 2008). Furthermore, if tetraploids are true hybrids, the question arises whether backcrosses between tetraploids and the two parental cytotypes are possible. Finally, the potential ability of these two species to hybridize raises the more general question of how genetically distinct they are from each other and what ecological and historical conditions have led to their differentiation. However, their genetic diversity has never been studied throughout their range up to date.

The aims of the present study were: (1) to investigate the variation in genome size within the two locations of sympatry between *R. nathaliae* and *R. serbica*, but also in several monospecific populations, to infer cytotype diversity; (2) to generate genetic profiles (using AFLP markers) of individuals within these two zones to test whether there is gene flow between the two species and to gain further insight into the genetic mechanisms that might explain the observed cytotype diversity; (3) to assess the genetic diversity and population structure of both species and their genetic differentiation using AFLP markers on a sample of monospecific populations covering most of their range in the Balkans. Knowledge of these genetic patterns is essential to provide a reference situation for the analysis of gene flow between the two species and especially in the two local areas of sympatry.

## Materials and Methods

### Plant Material

Analyses included two species of the family Gesneriaceae from the Balkan Peninsula, *Ramonda nathaliae* and *R. serbica* from monospecific populations, and a significant sample of individuals from the only two sites of sympatry known to date, both located in E Serbia (Table 1; Figure 1A). For chromosome counting, live plants were collected in the field and grown at the Institute of Botany and Botanical Garden “Jevremovac” in Belgrade and at the Laboratoire d’Ecologie, Systématique et Evolution à l’Université Paris-Saclay. For genome size estimation (GS), fresh leaves were collected in the field, brought to the laboratory in zip bags and stored in the refrigerator at 4°C until analysis. Leaves used for DNA extraction were collected directly in the field and immediately placed in silica gel-filled bags. Voucher specimens are deposited in the herbarium of the Institute of Botany and Botanical Garden “Jevremovac,” Faculty of Biology, University of Belgrade (BEOU).

**Table 1:** List of *Ramonda* populations included in the study. Dashed line represents the boundary between populations: used only for genome size estimation, or for two different molecular studies. In the last three columns, “1” indicates which analyses the individual populations were used for. Number of individuals included in molecular analyses is given in parentheses in the column AFLP.

Pop. code	Species	Locality	Altitude (m)	Latitude longitude	Geological substrate	Voucher number	Collectors	2*n*	2C	AFLP
N7	*R. nathaliae*	Serbia (Kosovo): Mt. Šara—Gotovuša	1,100	N 42°14′6.46″ E 21°4′30.14″	Limestone	21843 BEOU	Lazarević, M., Lazarević, P.		1	
N8	*R. nathaliae*	North Macedonia: Kozjak	1,610	N 41°23′56.72″ E 21°41′13.17″	Limestone	29886 BEOU	Niketić, M., Tomović, G.		1	
S11	*R. serbica*	Albania: Kruje	574	N 41°30′30.06″ E 19°47′45.13″	Limestone	32469 BEOU	Lakušić, D., Kuzmanović, N., Lazarević, P., Alegro, A.		1	
A	*R. nathaliae*	Serbia: Mt. Suva planina	1750	N 43°11′6.8″ E 22°10′0.7″	Limestone	20019 BEOU	Lakušić D.		1	1 (26)
B	*R. nathaliae*	North Macedonia: gorge of r. Treska, near Matka village	311.5	N 41°57′9.6″ E 21°17′51.5″	Limestone	31669 BEOU	Lazarević M., Sanino A., Siljak-Yakovlev S., Matevski V., Stevanović B., Stevanović V.		1	1 (26)
C	*R. nathaliae*	North Macedonia: gorge of r. Pčinja, near the road to Veles	266.1	N 41°49′18.9″ E 21°41′4.4″	Serpentine	31670 BEOU	Idem	1	1	1 (26)
D	*R. nathaliae*	North Macedonia: gorge of r. Vardar, near Demir Kapija	160	N 41°24′32.8″ E 22°15′42.1″	Limestone	31671 BEOU	Tomović G., Stevanović B., Stevanović V.			1 (26)
E	*R. nathaliae*	Greece: Mt. Vermion, gorge in the vicinity of village Sella, west of Noussa	*ca.* 1,000	N 40°35′5.4″ E 22°1′28.5″	Limestone	31668 BEOU	Niketić M.	1	1	1 (26)
F	*R. serbica*	Serbia: Radovanski Kamen, above the village Čukljenik	564	N 43°17′2.5″ E 22°4′16.4″	Limestone	31672 BEOU	Lazarević M., Lazarević P., Tomović G., Niketić M., Jović D., Petrović B.	1	1	1 (30)
G	*R. serbica*	Serbia: Mt. Rtanj	985	N 43°45′36.55″ E 21°55′40.01″	Limestone	31673 BEOU	Lazarević M., Lazarević P., Nešić D.		1	1 (8)
H	*R. serbica*	North Macedonia: gorge of r. Radika, Barička klisura	807	N 41°39′57.1″ E 20°37′3.6″	Limestone	Personal herbarium	Stevanović V., Tomović G.		1	1 (26)
I	*R. serbica*	Montenegro: gorge of r. Kroni e Murici	420	N 42°8′32.5″ E 19°13′9.9″	Limestone	19673 BEOU	Stevanović B., Stevanović V.	1	1	1 (26)
J	*R. serbica*	Montenegro: gorge of r. Kroni e Besit	250	N 42°9′19.7″ E 19°12′48.4″	Limestone	9880 BEOU	Stevanović B., Stevanović V.	1	1	1 (26)
K	*R. serbica*	North Macedonia: gorge of r. Crni Drim, near the village Lukovo	620	N 41°22′11.1″ E 20°35′57.6″	Limestone	2081 BEOU	Stevanović V., Tomović G.		1	1 (26)
L	*R. serbica*	Greece: Mt. Timfi, gorge of r. Vikos, near the village Vikos	*ca.* 650	N 40° 00′00″ E 20° 54′39.6″	Limestone	31674 BEOU	Niketić M., Tomović G.	1	1	1 (26)
M	*R. serbica*	Serbia: gorge of r. Mojstirska suhovara	920	N 42°55′39.6″ E 20°25′58.4″	Limestone	31675 BEOU	Lazarević M., Lazarević P., Pavićević D.		1	1 (26)
N	*R. serbica*	Serbia: gorge of r. Lazareva reka	310	N 44°1′38.1″ E 21°57′16.2”	Limestone	370/90	Lazarević M., Tomović G., Lakušić D.		1	1 (26)
O	*R. serbica*	Serbia: Čiflik	370	N 43°13′3″ E 22°25′6.2″	Limestone	31676 BEOU	Lazarević M., Lazarević P., Tomović G., Niketić M., Jović D., Petrović B.	1	1	1 (26)
P	*R. serbica*	Serbia: gorge of r. Godulja	750	N 42°55′27.4″ E 20°20′0.4″	Limestone	31677 BEOU	Lazarević M., Lazarević P., Pavićević D.		1	1 (5)
N3	*R. nathaliae*	North Macedonia: gorge of r. Vardar near Veles	165.8	N 41°41′15.2″ E 21°47′56.2″		33186 BEOU	Lazarević, M., Siljak-Yakovlev, S., Sanino, A., Matevski, V., Stevanović, B., Stevanović, V.		1	1 (25)
N5	*R. nathaliae*	Serbia: slopes of Mt. Suva planina near village Divljana in the vicinity of Bela Palanka	346.6	N 43°10′19.3″ E 22°18′10”	Limestone	20638 BEOU	Lazarević, M., Zlatković, B., Stevanović, V.	1	1	1 (17)
N6	*R. nathaliae*	North Macedonia: gorge of r. Raec near Kavadarci	295.7	N 41°26′14.1″ E 21°49′6.27″	Limestone	2063 BEOU	Lazarević, M., Šiljak-Yakovlev, S., Sanino, A., Matevski, V., Stevanović, B., Stevanović, V.		1	1 (25)
Nrk	*R. nathaliae*	Serbia: pure subpopulation from Radovanski Kamen	576.7	N 43°17′1.2″ E 22°4′23.5″	Limestone	20641 BEOU	Stevanović, V., Lazarević, M.	1	1	1 (16)
S1	*R. serbica*	North Macedonia: Galičica, Zli do	950	N 40°57′02.63″ E 20°48′02.73″	Limestone	Personal herbarium	Stevanović, B., Tomović, G., Stevanović, V.,			1 (24)
S7	*R. serbica*	Serbia: gorge of river Jelašnica in the vicinity of Niš	295.4	N 43°16′56″ E 22°3′42″	Limestone	20642 BEOU	Lazarević, M., Stevanović, V.	1	1	1 (24)
S9	*R. serbica*	Serbia: gorge of r. Nišava, 20 km east from Niš	308.5	N 43°18′38.1″ E 22°10′53.3″	Limestone	20639 BEOU	Lazarević, M., Zlatković, B., Stevanović, V.	1	1	1 (23)
S10	*R. serbica*	Serbia: gorge of r. Crna Reka in the vicinity of monastery St. Peter c. 8 km southwards from village Ribarići	1,100	N 42°56′46.3″ E 20°28′09.7″	Limestone	20623 BEOU	Lazarević, M., Lazarević, P., Pavićević, D.	1	1	1 (18)
Radovanski Kamen	Sympatry	Serbia: Radovanski Kamen	574.5	N 43°17′5.1″ E 22°4′16.6″	Limestone	20640 BEOU	Lazarević, M., Zlatković, B., Stevanović, V.	1	1	1 (55)
Oblik	Sympatry	Serbia: gorge of r. Nišava in the vicinity of village Ostrovica—Oblik	418.2	N 43°19′47.4″ E 22°8′44.9″	Limestone	20636 BEOU	Idem	1	1	1 (95)

### Chromosome Preparation and Estimation of Nuclear DNA Content by Flow Cytometry

Mitotic chromosome plates were prepared for two populations (one of *R. nathaliae* and one of *R. serbica*) as an addition to previously published chromosome numbers. This was done using pretreatment with 0.002 M 8-hydroxyquinoline for 4.5 h at 16°C, followed by cold fixation in 3/1 (v/v) ethanol/acetic acid for 24–48 h, hydrolysis for 14 min at 60°C, staining with Schiff reagent (Feulgen and Rossenbeck, 1924) and applying classical squash technique. Two to five individuals per population were used for chromosome counting.

Genome size was estimated for the first time in ten monospecific populations (four of *R. nathaliae* and six of *R. serbica*) as an addition to previously published results, as well as in two localities where these species grow in sympatry. Total nuclear DNA amount was determined by flow cytometry according to Marie and Brown (1993) on fresh leaves from at least five individuals per monospecific population (or three individuals in only two populations) and from 213 individuals from two hybrid zones. *Pisum sativum* cv. Long Express (2C = 8.37 pg) and *Lycopersicon esculentum* cv. Roma (2C = 1.99 pg) were used as internal standards. Nuclei were stained using propidium iodide, and measurements were done by Elite ESP flow cytometer (Beckman-Coulter, Roissy, France) equipped with argon laser. Both methods are described in detail by Siljak-Yakovlev et al. (2008).

### DNA Extraction and AFLP Analyses

AFLP analyses were performed by two independent molecular biology assays. A first experiment was designed to evaluate the genetic structure of populations of both species based on an initial sample of monospecific populations (16 populations in total—five of *R. nathaliae* and 11 of *R. serbica*, Table 1). This first analysis is referred to as the large-scale geographic study. The second AFLP experiment was above all designed to assess the hybridization potential of the two species when they occur in sympatry. Ten populations were studied: four monospecific populations of each species were included in this second analysis, as well as individuals collected at two sites of sympatry, Radovanski Kamen and Oblik (Table 1). Each individual from sites of sympatry was first identified as *R. nathaliae*, *R. serbica*, or “potential hybrid” based on its morphological characteristics (leaves and flowers). The material collected from individuals in sympatry consisted of leaves from 26 individuals potentially corresponding to *R. serbica*, 29 to *R. nathaliae*, and 40 individuals with ambiguous phenotypes at Oblik, and 11 individuals potentially corresponding to *R. serbica*, nine to *R. nathaliae*, and 35 individuals with ambiguous phenotypes at Radovanski Kamen. At each location of sympatry, sampling was conducted along a transect across the entire population. We systematically avoided sampling adjacent plants to eliminate the possibility of sampling clonal individuals since vegetative reproduction also occurs (Lazarević et al., 2013).

Fragments of leaves dried in silica gel weighting 21–26 mg were used for DNA extraction, which was performed using the NucleoSpin^®^96 Plant Kit (Macherey–Nagel) according to the manufacturer’s instructions. AFLP analyses were performed according to the method described by Vos et al. (1995) with some modifications (see Supplementary Material 1 for details).

Three primer combinations were selected for the full analysis (fluorescent dyes in parentheses): in both studies, EcoRI-AAC/MseI-CTC (FAM) and EcoRI-CAG /MseI-CTC (VIC) were used, whereas the third primer pair was different: EcoRI-ACA/MseI-CTC (NED) was used in the first AFLP experiment (monospecific populations only), and EcoRI-CAG/MseI-CTT (VIC) in the second experiment (hybridization study).

Amplified fragments were detected using the ABI Prism 3,100 sequencer on the Gentyane platform (INRAE, Clermont-Ferrand, France) and analyzed by GENESCAN 1.1 software (Applied Biosystems). DNA fragment detection results were indicated as present (1) or absent (0) by GeneMapper software version 4.0. Only unambiguous peaks in the size range of 50 to 500 bp were scored, with the minimum peak height set at 30. Each peak from each profile was checked by eye and its status changed accordingly. Finally, singletons (fragments that occur only in a single individual in the entire dataset) were removed to increase the reliability of the AFLP data.

To ensure the best possible reliability of AFLP markers, all samples from each AFLP experiment were managed in a single manipulation (same digestion, ligation, and PCR amplification mixtures and conditions for all samples). Two types of repeatability tests were performed. Four individuals, two from *R. nathaliae* and two from *R. serbica*, were used for the preliminary repeatability test. They were duplicated from the same amplification and then, genotyped with the same mixture of formamide and molecular weight marker. In this way, the reproducibility of the detection of the AFLP profiles was estimated (97.15%). In the second test, twenty individuals were amplified twice with two independent mixtures and the percentage of amplification reproducibility was 99.07%.

Because the samples from the two AFLP experiments were managed separately, the two datasets were not combined for the population genetic analyses to ensure better reliability of the analyses.

### Statistical Analyses and Population Genetics

Statistical analyses of genome size were performed in R4.1.2. For monospecific populations, each species was analyzed separately because genome size values differ greatly between the two *Ramonda* species. For small sample sizes, nonparametric tests were used (Fligner-Killeen test for homogeneity of rank variation and Kruskal–Wallis and Nemenyi tests for median comparisons). For pairwise comparisons, *p* values were corrected according to the Holm correction for multiple testing. Student t-test and Welch test (when homoscedasticity was rejected) were used for mean comparisons for larger sample sizes (*n* > 15).

AFLP-SURV 1.0 (Vekemans, 2002) was used to infer genetic diversity within populations. Allele frequencies were estimated from the presence or absence of fragment phenotypes using the Bayesian method described in Zhivotovsky (1999) with a non-uniform prior distribution of allele frequencies.

Genetic dissimilarity between individuals and populations was estimated using Shared Allele Distance (Chakraborty and Jin, 1993) with POPULATIONS 1.2.30 (Langella, 1999). Principal coordinate analysis (PCoA) was performed on the individual distance matrix. Neighbor-joining dendrograms were built using the population and individual distance matrices.

The distribution of genetic variation (between species, between populations, and within populations) was estimated by analysis of molecular variance (AMOVA) performed in Arlequin 3.5.1.2 (Excoffier and Lischer, 2010; http://cmpg.unibe.ch/software/arlequin35/Arlequin35.html). To test for correlations between geographic distance and genetic variance, a Mantel test was performed using the same software. The significance of *Φ*-statistics was tested based on the distributions of *Φ*-statistics calculated under the null hypothesis from 1,000 virtual samples obtained by random permutations of the original data set.

The genetic structure of the monospecific and the two populations of sympatry was studied using genetic model-based and non-genetic model-based methods. For the first type of analysis, Bayesian inference of genetic clusters and assignment of individuals was performed using STRUCTURE v.2.3.4 (Pritchard et al., 2000). STRUCTURE analyses were performed assuming admixture, correlated allele frequencies, and no prior population information. In a first step, 10 replicates were performed for each value of the genetic cluster (*K*), where *K* varied from 1 to 12, with a burn-in period of 50,000 iterations followed by a run length of 120,000 iterations of the Markov chain. The most probable number of groups was determined using the method of Evanno et al. (2005) implemented in Structure Harvester v.0.6.94 (Earl and vonHoldt, 2012). Then, 50 new replicates of the MCMC method were performed to determine the best K value. CLUMPP v.1.1.2 (Jakobsson and Rosenberg, 2007) was used to check for similarities between runs and average the membership probabilities. The final bar graphs showing the individual admixture coefficients were created using Structure Plot v.2.0 (Ramasamy et al., 2014).

In addition, and to evaluate the robustness of the classification used, a Discriminant Analysis of Principal Components (DAPC, Jombart et al., 2010) implemented in the adegenet package for R software was used for assigning each studied individual from the first study to a specific group based on its genetic characteristics.

Finally, in order to integrate cytogenetic data and genetic inferences, we provided distributions of ancestry coefficients values (*Q*-values) estimated by the Bayesian clustering analysis in sympatry and monospecific populations, according to ploidy levels inferred from genome size.

## Results

### Chromosome Counts and Genome Size in Monospecific Populations

As expected, chromosome number showed that all individuals sampled in monospecific populations of *R. nathaliae* were diploid with 2*n* = 2*x* = 48 chromosomes. The chromosome number of all individuals sampled in monospecific populations of *R. serbica* also confirmed that this species is hexaploid (2*n* = 6*x* = 144).

The average 2C DNA value was 2.33 pg in *R. nathaliae* and 7.83 pg in *R. serbica* (data from monospecific populations only; Table 2). Although differences in genome sizes between populations of *R. nathaliae* were significant (Kruskal–Wallis Chi-squared = 28.019, *df* = 8, *p* value < 1*e*-3), pairwise Nemenyi comparison tests yielded only three significant comparisons, all involving the population from Mt. Kozjak (N8) with slightly larger GS compared to other populations. There were also significant differences in genome size values between populations of *R. serbica* (Chi-squared = 40.405, *df* = 13, *p* value < 1*e*-3). However, this was the result of differences between populations with the smallest (Mt. Timfi and Lazareva Reka) and populations with the largest (Nisava and Crna Reka) genome size values.

**Table 2:** Chromosome numbers and nuclear DNA contents of investigated monospecific populations of *R. nathaliae* and *R. serbica* (*N* = number of samples for 2C DNA value).

Pop. Code	Species	Locality	*N*		2*n*	Ploidy level (*x*)	2C in pg and (Mbp)^a^	CV (%)	1Cx in pg and (Mbp)
A	*R. nathaliae*	RS: Mt. Suva planina	5			2	2.28 (2229)^*^	2.34	1.14 (1115)
B	*R. nathaliae*	MK: Matka	5			2	2.26 (2208)	1.50	1.13 (1104)
C	*R. nathaliae*	MK: Pčinja	5		48* *	2	2.27 (2217)^**^	1.54	1.13 (1108)
E	*R. nathaliae*	GR: Mt. Vermion	5		48	2	2.28 (2231)^*^	1.93	1.14 (1116)
N3	*R. nathaliae*	MK: Vardar – Veles	5			2	2.35 (2295)^**^	0.50	1.17 (1147)
N5	*R. nathaliae*	RS: Divljana	5		48*	2	2.33 (2277)^*^	1.95	1.16 (1138)
N6	*R. nathaliae*	MK: Raec – Kavadarci	5			2	2.37 (2318)	0.88	1.18 (1159)
N7	*R. nathaliae*	RS (XK): Gotovuša	5			2	2.32 (2266)	2.17	1.16 (1133)
N8	*R. nathaliae*	MK: Kozjak	5			2	2.50 (2445)	0.92	1.25 (1222)
	*R. nathaliae*	Mean value	45			2	2.33 (2278)	3.36	1.16 (1139)
G	*R. serbica*	RS: Mt. Rtanj	5			6	7.76 (7587)	1.04	1.29 (1264)
H	*R. serbica*	MK: Radika	5			6	7.88 (7706)	0.77	1.31 (1284)
I	*R. serbica*	ME: Kroni e Murici	5		~150^*^	6	7.94 (7763)*	2.38	1.32 (1294)
J	*R. serbica*	ME: Kroni e Besit	5		144^*^	6	7.77 (7601)*	0.50	1.30 (1267)
K	*R. serbica*	MK: Crni Drim	5			6	7.95 (7772)	0.32	1.32 (1295)
L	*R. serbica*	GR: Mt. Timfi	5		144^**^	6	7.58 (7416)^**^	2.29	1.26 (1236)
M	*R. serbica*	RS: Mojstir	5			6	7.91 (7737)	2.49	1.32 (1289)
N	*R. serbica*	RS: Lazareva reka	5			6	7.58 (7413)^*^	1.37	1.26 (1236)
O	*R. serbica*	RS: Čiflik	5		144	6	7.79 (7619)	2.92	1.30 (1270)
P	*R. serbica*	RS: Godulja	5			6	7.61 (7446)^**^	6.28	1.27 (1241)
S7	*R. serbica*	RS: Jelašnica	5		144^**^	6	7.85 (7678)^**^	0.86	1.31 (1280)
S9	*R. serbica*	RS: Nišava	5		> 144^*^	6	8.11 (7927)^*^	1.86	1.35 (1321)
S10	*R. serbica*	RS: Crna Reka	3		144^*^	6	8.32 (8136)^*^	1.82	1.39 (1356)
S11	*R. serbica*	AL: Kruje	3		-	6	7.76 (7592)	1.04	1.29 (1265)
	*R. serbica*	Mean value	66			6	7.83 (7660)	3.15	1.31 (1277)

a Values in Mbp calculated based on *pg* = 978 Mbp *(*Doležel et al., 2003).

* *2*C values and/or chromosome numbers from Siljak-Yakovlev et al. (2008);

** *2*C values and/or chromosome numbers from Lazarević et al. (2013).

### Large-Scale Geographic Study of Genetic Diversity in Their Entire Range

#### Genetic Diversity Revealed With AFLP Markers

In the first molecular study, a total of 1,077 AFLP fragments were obtained with the three primer pairs. Once singletons excluded 163 fragments were present only in *R. nathaliae,* and 235 fragments were private to *R. serbica*. However, none of these fragments was fixed for alternative alleles in the two species (fragments present in all individuals of one species and absent in all individuals of the other species). Low frequency fragments dominated in both species. The number of fragments detected was moderately higher in *R. serbica* than in *R. nathaliae* (938 in the former and 866 in the latter), a trend expected due to the larger genome size of *R. serbica*.

Within-population genetic diversity was moderately similar in *R. serbica* (mean *Hj* = 0.105) and *R. nathaliae* (mean *Hj* = 0.092; Supplementary Table 1). Very little variation from the expected heterozygosity was observed between populations of each species. The lowest genetic diversity was found in *R. nathaliae* from Mt. Suva Planina in E Serbia (A), and the highest values were found in *R. nathaliae* from Matka in North Macedonia (B) and *R. serbica* from Mt.Timfi in Greece (L).

#### Genetic Differentiation and Population Genetic Structure

**Figure 2:**
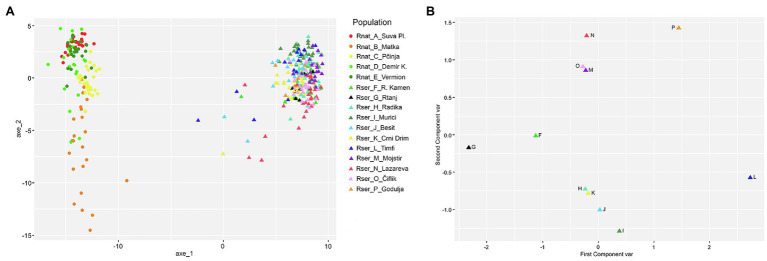
Principal Coordinate analysis performed on the large geographical scale AFLP dataset using, the Shared Allele Distance computed on: **(A)** individuals of both *R. serbica* and *R. nathaliae* species (the first two axes represented 48.8 and 4.0% of the total variation, respectively); **(B)** on populations of *R. serbica* alone (the first two axes represented 52.2 and 30.0% of the total variation, respectively).

AMOVA and PCoA analyses showed that most of the genetic diversity within the entire sample are explained by genetic differentiation between the two species, as would be expected in the case of truly independent evolutionary lineages (*Φ*ct = 0.718, see Supplementary Table 2; Figure 2A). The genetic differentiation of populations within species is also very high (*Φ*sc = 0.318). However, AMOVA analyses for individual species showed that population genetic differentiation was much higher in *R. nathaliae* (*Φ*st = 0.432) than in *R. serbica* (*Φ*st = 0.244).

Total genetic diversity (Ht) was equally low in both species with values of Ht = 0.116. Among the populations of *R. nathaliae*, the lowest genetic distance was observed between the populations of Mt. Vermion (E) and Demir Kapija (D) and the highest between Mt. Suva Planina (A) and Matka (B) (Supplementary Figure 1). In *R. serbica*, a population from Mt. Timfi (L), at the southern edge of the species’ range, and Godulja (P) in SW Serbia, were genetically the most distant from the others (Supplementary Figure 1). PCoA analysis of populations of *R. serbica* revealed a clear geographic pattern of genetic differentiation, contrasting northern populations from Serbia (G, F, P, N O, and M) on one side and southwestern populations from Greece, North Macedonia, and Montenegro (I, J, H, K, and L) on the other (Figures 1A, 2B). The relationship between pairwise geographic distances between populations and genetic distances was not significant for *R. nathaliae* (Z = r = 0.51; *p* = 0.131), but was significant in *R. serbica* (Z = r = 0.56; *p* < 1e-3). However, this relationship is mainly due to the differentiation between northern and southern populations of this species. Overall, geographic distance between populations explained their genetic distances poorly, as can be clearly seen for northern populations G and F on the one hand, and M, N, O, and P on the other.

DAPC analysis also showed that the vast majority of individuals of both species could be unambiguously assigned to their population of origin (Supplementary Figure 2). There were only a few cases of assignment uncertainty or assignment to a different population in *R. serbica* between two populations in E Serbia (Radovanski Kamen (F) and Mt. Rtanj (G)) that were only 50 km apart, and also between two geographically very close populations (I and J), both from Mt. Rumija in Montenegro.

Interestingly, a few *R. serbica* individuals from several populations (F, J, K, L, and N) showed a slightly intermediate genetic profile between the two species (Figure 2A), although they were well assigned to their own species in the DAPC analysis.

### Evidence for Hybridization in Two Sites of Sympatry

#### Genome Size Variability in the Two Sites of Sympatry

**Figure 3:**
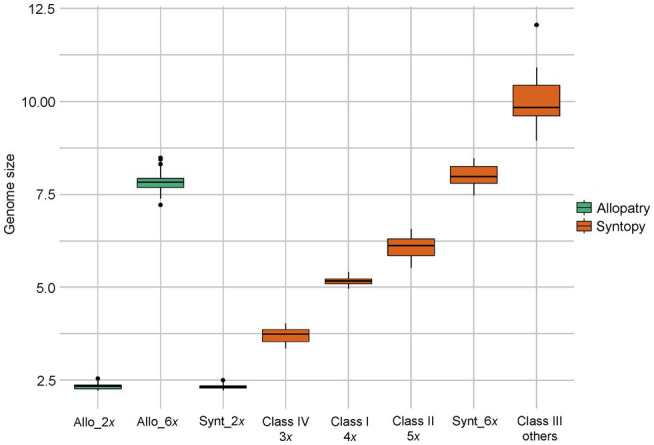
Box-plot showing the distribution of genome size of each taxon and each genome class (according to Table 3).

Individuals from both locations of sympatry showed tremendous but discontinuous variability in genome size (Table 3; Figure 3). A first group of individuals had a mean genome size of 2.32 pg (range between 2.22 and 2.51 pg). The two mean GS values of this group in Oblik and in Radovanski Kamen were not significantly different (Student-*t* = 1.79, df = 73, *p* value = 0.078). The GS of this group was also not significantly different from the mean genome size of *R. nathaliae* estimated from monospecific populations (Student-*t* = 0.50, df = 118, *p* value = 0.615). Thus, the plants in this group certainly corresponded to diploid individuals.

**Table 3:** Chromosome numbers, nuclear DNA contents and ploidy levels of individuals from sympatry between *R. nathaliae* and *R. serbica* (*N* = number of samples for 2C DNA value).

Locality	Taxon	Ploidy level (*x*)	2*n*	2C (pg)	CV (%)	Min–Max of 2C (pg)	*N*
RS: Oblik	*R. nathaliae*	2	–	2.34	2.00	2.27–2.45	30
RS: Radovanski Kamen	*R. nathaliae*	2	48^*^	2.31	2.81	2.22–2.51	45
RS: Oblik	*R. serbica*	6	–	7.95	2.01	7.72–8.29	23
RS: Radovanski Kamen	*R. serbica*	6	144^*^	8.00	4.38	7.48–8.45	18
RS: Oblik	Class I	4	96^*^	5.14	1.41	5.03–5.27	25
	Class II	–	–	6.12	–	–	1
	Class III	–	–	10.32	12.83	8.94–12.06	4
RS: Radovanski Kamen	Class I	4	96^*^	5.18	1.98	4.96–5.39	45
	Class II	–	–	6.05	5.25	5.52–6.55	14
	Class III	–	–	9.98	5.14	9.54–10.88	6
	Class IV	–	–	3.70	8.97	3.35–4.01	3

Individuals whose genome size did not correspond to those of either *R. nathaliae* or *R. serbica* were classified into four different classes.

* Chromosome numbers from Siljak-Yakovlev et al. (2008).

A second group of individuals had a mean genome size of 7.98 pg (range between 7.48 and 8.45 pg). This group showed no significant difference between Oblik and Radovanski Kamen (Welch-t = −0.067, df = 22.53, p value = 0.96). Although the mean GS of this group at the two sites was significantly higher than the mean of the monospecific populations of *R. serbica* (Student-t = −2.851, df = 105, p value = 0.005), it remained within the range of variation of *R. serbica*. Pairwise comparisons showed that only the comparison with the Lazareva Reka population was at the threshold of significance (data not shown). Therefore, this group of individuals was likely hexaploid at both sites of sympatry.

However, other classes of genome size values suggest that there are several other ploidy levels at both sites of sympatry with genome composition contributed by both species. Indeed, a first class of individuals had a mean GS value of 5.17 pg (range between 4.96 and 5.39) (*cf.* Class I in Table 3). The individuals of this class in Oblik and Radovanski Kamen did not show any significant difference in their mean GS (Student-t = −1.65, df = 68, p value = 0.104). The mean GS of this entire class was very close, although significantly higher (Student-t = 7.79, df = 69, p value = 4.8e-11) than the expected value for a first-generation tetraploid interspecific hybrid (5.08 pg) calculated from the mean GS values of the two *Ramonda* species. This supported that these individuals are interspecific tetraploid hybrids. It is noticeable that a few individuals exhibited a combination of morphological characteristics of the two species (Figure 1B), suggesting that they may be first-generation interspecific hybrids. These plants were very abundant in our sample, representing 70 out of 214 individuals analyzed in the two sites of sympatry.

Finally, two other classes of GS values were also observed in the two sites of sympatry. Fifteen analyzed individuals had a higher genome size than the hypothetical tetraploid hybrids, ranging from 5.52 pg to 6.55 pg (*cf.* Class II in Table 3, Figure 3). In some of these individuals, the GS was close to what would be expected in a 5*x* interspecific hybrid. For example, a 5*x* individual with two *R. nathaliae* genome complements and three *R. serbica* genome complements would be expected to have a GS of about 6.27 pg. Thus, the plants in this Class II could correspond to individuals with mosaic genomes in which the proportion of the two species is different than in a first-generation tetraploid hybrid.

In both populations, a few individuals (10 in total) had a higher genome size than the hexaploid *R. serbica* (from 8.94 to 12.06 pg, *cf.* Class III in Table 3).

Finally, three individuals sampled at Radovanski Kamen were found to have an intermediate genome size between *R. nathaliae* and tetraploid interspecific hybrids (Class IV, Table 3). Their GS values were close to what would be expected in triploid individuals of *R. nathaliae*.

Taxonomic identification of individuals in monospecific populations of the two *Ramonda* species based on leaf and flower morphological characters is always trivial and unambiguous (Siljak-Yakovlev et al., 2008; Lazarević et al., 2013, 2014). However, this was not the case for all individuals sampled at the two sites of sympatry (Figure 1B). For this reason, the GS values were also used to confirm or assign a taxonomic identification to be used in the analyses of the AFLP data (see below). In the following, all individuals in sympatry with the same genome size as *R. nathaliae* were classified as this species, and the same principle was applied to *R. serbica*. Other individuals are hereafter referred to as potential “hybrids” and assigned to their GS classes as indicated in Table 3.

#### Genetic Variability by AFLP Markers in the Two Sites of Sympatry

The following analyses included eight monospecific populations (four for each species) that were used as references in addition to the individuals sampled at the two sites of sympatry (Table 1). After removing singleton fragments, 1,263 AFLP fragments remained for analyses. None of the fragments were present in all individuals of one species and absent in all individuals of the other species, similarly to what was found in the large-scale geographic study. However, some fragments were highly abundant in one species and absent in the other, consistent with the data from the first set of populations (see above).

A strong contrast in AFLP fragment frequencies between *R. serbica* and *R. nathaliae* would have been expected because of the genome size and ploidy level of *R. serbica*. In fact, the distribution of the difference in fragment frequencies between the two species was clearly centered at zero (median = −8e-4; mean = 0.049) (Supplementary Figure 3). Thus, the dominant alleles at the AFLP markers (presence of the fragment) were almost equally distributed between the two species. Importantly, this result supports the assumption that in this study the dominance effect at AFLP markers was unlikely to cause bias in favor of either species in individual assignment in subsequent clustering analyses.

**Figure 4:**
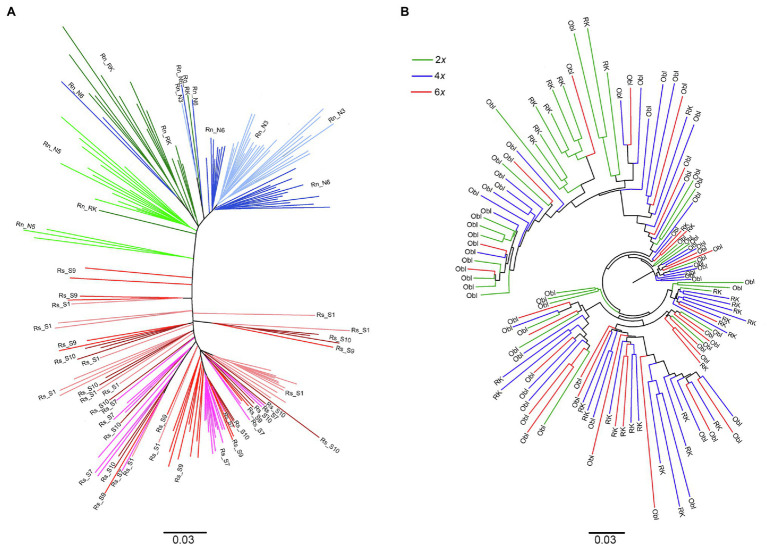
Dendrogram built on inter-individual Shared Allele Distance using: **(A)** individuals of *R. serbica* and *R. nathaliae* sampled in allopatric monospecific populations (each color indicates a specific population whose code names are referred to in Table 1); **(B)** individuals from the two sites of sympatry [Oblik (Obl) and Radovanski Kamen (RK)]. All those individuals contributed to the interspecific gene flow study.

The strong genetic differentiation between the two species, already reported in the large-scale geographic study, was confirmed when only monospecific populations were considered (*Φ*ct = 0.548; Figure 4A). In contrast, it was noticeable that no genetic differentiation (*Φ*ct = −0.009) was observed between diploid and hexaploid individuals from the two sites in sympatry, as also evidenced by the strong mixing of individuals from both ploidy levels in the dendrogram (Figure 4B). The tetraploid plants also exhibited similar genetic variability as the diploid or hexaploid plants in sympatry as shown by the very strong mixing of the three ploidy levels in Figure 4B. However, clear and even strong genetic differentiation was observed between individuals from the two sites of sympatry for each ploidy level (*Φ*st = 0.22 for diploids and *Φ*st = 0.10 for hexaploids). Consistent with this result, clusters of individuals from each location of sympatry, regardless of their ploidy level, are clearly visible in Figure 4B.

**Figure 5:**
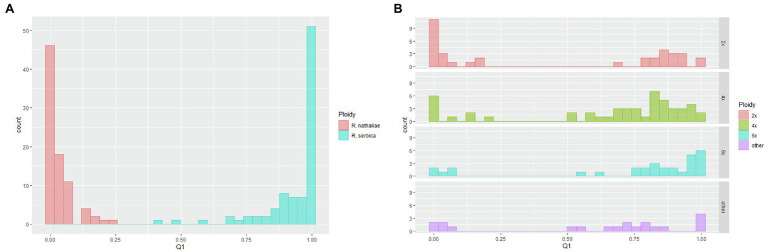
Distribution of individual ancestry coefficients (inferences from the Bayesian clustering analysis) to the “serbica” genetic cluster for: **(A)** *R. nathaliae* (red) and *R. serbica* (blue) allopatric populations; **(B)** for individuals sampled in the two sites of sympatry, represented according to their genome size. “Other” refers to individuals that could not be undoubtedly assigned to a given ploidy level from their genome size.

Bayesian clustering yielded two genetic clusters as the solution that best fit the data, according to the Evanno test (Supplementary Figure 4) and the very clear plate obtained by the likelihood of the data at *K* = 2 (data not shown). This result was to be expected, since species differentiation is the factor that contributes most to the genetic structure of the population in the entire data set. Indeed, from the data of monospecific populations, it appears that the two genetic clusters correspond to one of each species (Supplementary Figure 5). DAPC analysis confirmed this result. Indeed, although *K* = 3 was the optimal solution (Supplementary Figure 6), one of those two clusters was composed only with one individual of the S7 monospecific population of *R. serbica* (Supplementary Table 3). As for the Bayesian clustering analysis, the two main clusters corresponded to *R. nathaliae* and *R. serbica*. We will therefore refer to “nathaliae” and “serbica” for these two clusters below. The distribution of individual ancestry coefficients showed two very different patterns between the monospecific populations and sites of sympatry (Figure 5, Supplementary Figure 5). Most individuals in monospecific populations had high ancestry values of the species where they belonged (mean ancestry of 0.93 for individuals of *R. serbica* to the genetic cluster “serbica” and 0.96 for individuals of *R. nathaliae* to the genetic cluster “nathaliae”; Figure 5A). Therefore, the AFLP data allowed unambiguous assignment of nearly all individuals sampled in monospecific populations to their own species, as already observed in the large-scale geographic study. Only three individuals of *R. serbica*, all from the gorge of Nišava river (S9), had an intermediate ancestry coefficient (Figure 5A), with the ancestry coefficient to the “serbica” genetic cluster ranging from 0.43 to 0.59. In comparison, the lowest value of ancestry to the “nathaliae” genetic cluster was up to 0.75 for individuals sampled in monospecific populations of *R. nathaliae*.

The two sites of sympatry showed a very different and more complex genetic pattern, similar to what was observed for genome size. The main difference with the monospecific populations was that there was much less agreement between the genome size values (and thus the inferred ploidy level) and the AFLP profiles. In fact, when 2*x* and 6*x* individuals were considered, the distribution of ancestry coefficients was bimodal for both cytotypes (Figure 5B). Surprisingly, 17 of the 35 diploid individuals had high ancestry coefficients to the hexaploid *R. serbica* (mean = 0.88; range: 0.68–0.99 for these 17 individuals). All but one of them were sampled in the Oblik site of sympatry. The remaining 18 diploid individuals had very high ancestry coefficients to *R. nathaliae*, as expected based on their assumed ploidy level (mean = 0.96; range: 0.83–1). In contrast, among hexaploid individuals, five of the total 30 individuals, all from Oblik, showed strong ancestry to *R. nathaliae*, contrary to what was expected based on their genome size.

The bimodal character of the distribution of the ancestry coefficient distribution was also observed for tetraploid individuals (Figure 5B), although the right side of the distribution was much flatter than that observed for monospecific *R. serbica* populations. This also testifies to the high genetic heterogeneity of tetraploid plants. The mean ancestry value of the 40 individuals belonging to the right side of the distribution was 0.80, very close to the value of 0.75 expected in the case of a first-generation hybrid resulting from a cross between hexaploid *R. serbica* and diploid *R. nathaliae*. However, ten individuals, all from Oblik (Supplementary Figure 5), formed the left side of the distribution (i.e., individuals unambiguously assigned to *R. nathaliae* based on AFLP markers). Their mean ancestry value to “serbica” genetic cluster was 0.05 (range: 0.01–0.2), very close to the value obtained for *R. nathaliae* individuals from monospecific populations.

Apart from these three groups of individuals, eight individuals from Radovanski Kamen had rather mixed genetic profiles with a GS lower than *R. serbica* but higher (between 5.61 and 6.55 pg) than potential 4*x* individuals (*cf.* Class II in Table 3), but with a mean ancestry coefficient of 0.78 (range between 0.56 and 0.99) much closer to *R. serbica* than to *R. nathaliae*. Of the nine individuals in the Class III of genome size (ranging from 8.94 to 10.5 pg, Table 3), three were strongly assigned to the *R. serbica* genetic cluster, while two had a very high ancestry coefficient to *R. nathaliae*. This last observation suggests the existence of autopolyploid individuals with high ploidy level descended from *R. nathaliae*. Finally, the three individuals with a genome size between 3.35 and 4.01 pg (i.e., a GS between 2*x* and 4*x*) were unambiguously assigned to *R. nathaliae*.

It is noticeable that individual assignment posterior probabilities inferred by the DAPC analysis and ancestry coefficients inferred through the Bayesian approach were highly correlated (0.95 for all individuals in sympatry and 0.96 for the whole sample).

Both GS and AFLP patterns showed that more complex genomic structures than first-generation interspecific tetraploid hybrids are present at both sites of sympatry.

## Discussion

### Chromosome Number, Genome Size, and Ploidy Level in Allopatric Populations

In the past, there were uncertainties concerning the chromosome number and ploidy levels of Balkan *Ramonda* species. Although Glišić (1924) found *n* = 36 to be the most frequent haploid chromosome number in *R. nathaliae*, later both Ratter (1963) and Contandriopoulos (1966) showed that *R. nathaliae* is a diploid with 2*n* = 2*x* = 48 chromosomes, and this is confirmed by broad and detailed cytogenetic studies by Siljak-Yakovlev et al. (2008) and Lazarević et al. (2013). Still, some uncertainties remained about chromosome number and ploidy level in *R. serbica* based on the following reports: 2*n* = 72 in *R. serbica* individuals collected in Sićevo Gorge in E Serbia (Glišić, 1924), 2*n* = 96 in plants from Mt. Timfi—Vikos Gorge in Greece (Contandriopoulos, 1966), polyploid forms as a mixture of several chromosome numbers, from less than 72 to more than 144 chromosomes, with 2*n* = 72 and 2*n* = 96 being most common in only two populations in Albania (Daskalova et al., 2012). In this last study, five individuals per population were analyzed and hexaploid individuals were not detected. However, one of the populations analyzed for genome size in the present study is Kruje, the same population as in Daskalova et al. (2012), and only 6*x* individuals were found. In the last 15 years, Siljak-Yakovlev et al. (2008) and Lazarević et al. (2013), as well as the present study, have analyzed the genome size and/or chromosome number of a total of 133 individuals sampled from 16 monospecific populations of *R. serbica* throughout the species range. The vast majority of them were hexaploid individuals with 2*n* = 6*x* = 144 chromosomes. Only in one population from Montenegro were two individuals found with a larger genome size, suggesting the possibility of the existence of 8*x* and even 10*x* individuals (Siljak-Yakovlev et al., 2008). All this indicates possible but rare alternative chromosome numbers in monospecific populations.

### Genetic Diversity in *Ramonda* Species From the Balkan Peninsula

To date, very few studies have examined the genetic diversity of the two Balkan *Ramonda* species. In a study on the genetic variability of three populations of *R. serbica* from Albania and three populations of *R. nathaliae* from North Macedonia using RAPD markers, Daskalova et al. (2012) could not clearly distinguish the two species. More recently, Petrova et al. (2018) studied five populations of *R. serbica* in Bulgaria using ISSR markers. They showed that this species has low genetic variability and significant differentiation among populations. The present study is the first investigation of the genetic diversity of *R. nathaliae* and *R. serbica* as a whole, using the same molecular markers and including a large coverage of their respective ranges in the Balkan Peninsula. The number of AFLP markers is also high, and thus, we are confident that genetic diversity and structure patterns here reported are representative. The high level of genetic differentiation is in agreement with morphological data that have shown that the two species are broadly consistent across their range, with the exception of the two sites of sympatry (Lazarević et al., 2013, 2014). Therefore, it is clear that the two species actually correspond to two independent evolutionary lineages, suggesting a unique origin of hexaploid monospecific populations in their range. Moreover, both species showed a similar picture: a very high level of genetic differentiation between populations within species and very low signals of genetic admixture between populations of each species, except between a few pairs of geographically close populations.

What information about the evolutionary forces that shaped genetic diversity in these two species can be inferred from this pattern? There is no doubt that the dynamics and extent of the glacial periods in the mountains of the central part of the Balkan Peninsula contributed to the genetic and chorological differentiation of these two species. Glaciation in this region was much less pronounced than in other parts of Europe (Hughes et al., 2006), raising the possibility of existence of multiple and scattered refugia for plant and animal species survival in the Balkan region (Hewitt, 1996). Available data show that the snowline in different parts of the Mediterranean region, and especially in the Balkan Peninsula, extended to different heights, from down to 1,250–1,600 m in the highest mountains of the region (Menkovic et al., 2004; Hughes et al., 2006), to 270 m in some coastal parts of the Dinarides (Marjanac and Marjanac, 2004). There is a possibility that the Balkan *Ramonda* species were mountain plants that descended to refugia in the surrounding gorges and canyons during the Ice Age, where they are still predominantly found today (Košanin, 1921; Stevanović et al., 1986; Lazarević, 2012). Also, the occurrence of a series of lakes during the Miocene and Pliocene, which spread in different parts of the Balkan Peninsula (Krstić et al., 2012), may have had a significant impact on the distribution of plant species and contributed to the isolation of *Ramonda* populations from each other.

Thus, the strong genetic differentiation within species and between populations exhibited by the two *Ramonda* species is likely the result of long reproductive isolation of populations and strong drift effects during glacial periods, but possibly before as well (Krstić et al., 2012), as the geographic distribution of populations is highly fragmented. A similar pattern was found for *R. myconi* in N Spain and the central Pyrenees, corresponding to its entire range, with high genetic differentiation between regions (Dubreuil et al., 2008). This pattern led the authors to suggest that this species occurred in several different but genetically unrelated refugia in this area. Similarly, in another Tertiary relict species from the family Gesneriaceae *Haberlea rhodopensis* Friv. two distinct genetic clusters were identified in Bulgaria, and the existence of which was suggested to be a consequence of isolation between refugial populations (Petrova et al., 2014). Finally, Petrova et al. (2018) have shown that the genetic diversity of *R. serbica* populations in Bulgaria is very low. The authors hypothesize that this is the result of both post-glacial fragmentation and a particularly pronounced lack of genetic connectivity for the Bulgarian populations, which are on the periphery of the species range.

This pattern of variability is expected in long-living plants with geographically isolated populations and probably with limited seed dispersal, as is the case in *Ramonda* species (Picó and Riba, 2002; Lazarević et al., 2013; Petrova et al., 2014). The overall genetic diversity of the two studied species is low, similar to what was found for *R. myconi* in the Pyrenees (Dubreuil et al., 2008) and *H. rhodopensis* in Bulgaria and Greece (Petrova et al., 2014, 2015). In the post-glacial period, eventual dispersal outside refugial areas may also have led to repeated demographic bottlenecks, which should enhance or at least maintain genetic differentiation of populations. All the more, these two species still have a very patchy distribution and most populations are still spatially isolated. It is noticeable that the population of *R. nathaliae* with the lowest genetic diversity is found on Mt. Suva Planina, which is at the highest altitude of all populations of this species studied here. Living conditions are harsh there, and rosettes are much smaller than elsewhere (authors’ personal observation). It is therefore likely that the very low genetic diversity of this population is the result of a very small effective size, due to the combined effects of small size and potential selection.

The current ranges of these two species do not overlap, and only two narrow areas of sympatry have been observed so far (Stevanović et al., 1986). This observation and the marked genetic differentiation between these two species may indicate both that the two species were separated prior to the ice ages and that their refugia were distant from each other. However, despite the high genetic differentiation between the two species, no specific markers (alternative alleles fixed in either species) were found among the hundreds of AFLP fragments revealed in this study. However, this result should be confirmed on the basis of orthologous sequence data. The absence of specific markers may be evidence of a relatively recent divergence between the two species, which is consistent with the results of Petrova et al. (2015) on Gesneriaceae species. Based on chloroplastic and ITS sequence polymorphisms, these authors inferred that the timing of the divergence between *R. nathaliae* and *R. serbica* may have occurred in the middle or last Pleistocene. This divergence is one of the youngest divergence times among Gesneriaceae species estimated in this study. This could also mean that the genus *Ramonda* is a relict of the Tertiary, but that the individual species are actually younger.

### Interploidy Gene Flow

Polyploidy is common in plant species and is mainly favored by meiosis defects leading to the production of unreduced gametes (Brownfield and Köhler, 2011; Mason and Pires, 2015; Kreiner et al., 2017). Intrageneric variability in ploidy level is widespread in the literature, and situations have been described where different cytotypes coexist in large contact zones as well as in geographically much narrower areas. Therefore, the possibility of interploidy gene flow within the same species or between related species has been a focus of attention for many years. However, the vast majority of cases reported in the literature in natural species involved diploid and tetraploid cytotypes, whereas cases of contact zones between diploid and hexaploid plants have been reported less frequently (see Kolář et al., 2017 for a comprehensive review of reported cases). In *Aster amellus* L., for example, diploid and hexaploid cytotypes are usually found in separate locations, while a few parapatric and one mixed-ploidy population have been observed. Based on fine-scale cytotype screening, the authors concluded that there is no evidence for the existence of tetraploid plants (i.e., first-generation hybrids) probably as a consequence of the exclusion of minority cytotypes (Castro et al., 2012), as well as niche differentiation and shifts in flowering times between cytotypes (Raabová et al., 2008). One of the most comparable situations to that observed in Balkan *Ramonda* is the case of *Senecio carniolicus* Willd. (syn. *Jacobaea carniolica* (Willd.) Schkrank), an autopolyploid complex with diploid and hexaploid plants as the most widespread ploidy levels. In contrast to *Ramonda* species, a high number of mixed ploidy populations were found in this species (Sonnleitner et al., 2010). Tetraploid individuals were observed in restricted areas, which the authors believe may correspond to glacial refugia in the eastern Alps. Other ploidy levels (3*x*, 5*x*, 7*x*−9*x*) are also observed, but at very low frequency. The authors noted that there should be barriers limiting the production and/or maintenance of interploid hybrids, to which the distinction between 2*x* and 6*x* habitats contributes. Lower fitness of progeny from crosses between diploids and polyploids also clearly contributes to limited gene flow between cytotypes (Sonnleitner et al., 2013).

It is necessary to emphasize that in the case of *Ramonda*, in contrast to the examples cited above, it is a question of two cytotypes (2*x* and 6*x*) belonging not to one but to two different species. This makes hybridization more difficult and the genome constitution of the hybrids extremely diverse and sometimes difficult to explain. The case of *Ramonda* documented in this article therefore stands out from most studies of mixed-ploidy populations. It is interesting because, first, it shows enormous variability in genome size at micro-level in the only sites of sympatry between two species. This variation in GS allows us to hypothesize that the observed ploidy levels are also diverse, ranging from at least 2*x* to 8*x* (or possibly up to 10*x*), including odd ploidy levels such as at least 3*x* and 5*x*. For example, the class III of genome size (range between 8.94 and 12.06 pg, Table 3) could include 7*x*, 8*x*, but also higher ploidy levels. Based on the mean 1C*x* values of *R. nathaliae* and *R. serbica*, one would expect GS values between 8.15 and 9.24 pg for 7*x* individuals. For 8*x* individuals, the expected range of variation would be between 9.32 and 10.56 pg. Therefore, we could not exclude the possibility that 9*x* and/or 10*x* ploidy values were also present in our sample.

Octoploid individuals may have arisen from whole genome duplication (WGD) of tetraploid individuals, resulting in potential stabilization of the hybrid genome, a phenomenon already suggested by several authors (reviewed in Glombik et al., 2020). In contrast, the extended study of genome size in the two Balkan *Ramonda* species reported here showed that there was no cytotype variability within each species in monospecific populations, except for two individuals of *R. serbica* from Montenegro that were presumably 8*x* and 10*x* (Siljak-Yakovlev et al., 2008). Aneuploidy could also contribute to the observed variation in GS in both sites of sympatry. However, these hypotheses remain to be confirmed by cytogenetic studies. Finally, genome rearrangements and transposition activation have been documented in artificial interspecific hybridization experiments (Szadkowski et al., 2011; Liu et al., 2014; Mason and Wendel, 2020). We cannot rule out that such phenomena also contributed to the large variation in genome size observed in both sites of sympatry.

Nevertheless, the proportion of cytotypes distinct from the canonical ploidy levels 2*x* and 6*x* (a total of 33% of 4*x* plants) appears to be very high in these two sites of sympatry compared with most previously reported studies (Kolář et al., 2017). Other classes of cytotypes were also not rare [for example, 6% of plants suspected of being 5*x* (referred as class II in Table 3)].

The existence of hybridization between the two species, *viz.* between 2*x* and 6*x* cytotypes, in each of the two sites of sympatry was clearly demonstrated by: (i) the presence of individuals with an intermediate genome size compatible with the expected value for F1 tetraploid hybrids given the genome sizes of the two parental species; (ii) the presence of tetraploid individuals combining morphological features of the two species; and (iii) the observation that diploid and hexaploid individuals at the two sites of sympatry differed genetically much less (based on AFLP markers) than in monospecific populations. Several factors make this hybridization between the two species in sympatry possible. Both species flower at the same time. Although the flowers are slightly different, they are most likely pollinated by the same pollinators (Lazarević et al., 2013). Our results showed therefore that pre- and/or post-zygotic barriers to hybridization that usually exist between different cytotypes and/or plant species (Vallejo-Marín and Hiscock, 2016) are not operative in two sites of sympatry (Mallet, 2007; Abdelaziz et al., 2021).

The joint distribution of cytotypes and ancestry coefficients estimated from AFLP data in the two sympatry sites revealed a very singular pattern, namely a bimodality of these coefficients for diploids and hexaploids, compared to that observed in allopatric monospecific populations, but also for tetraploids. This striking observation may help to better understand the evolutionary dynamics that took place at these two sites by suggesting hypotheses on the pathways by which cytotype diversity was achieved and on the processes that might have been involved in the maintenance of tetraploid individuals and that would explain their genetic constitution.

The high number of tetraploid plants and their high AFLP diversity, similar to that of diploid and hexaploid plants in both sites of sympatry, suggests that hybridization between the two species is not an uncommon event in these sites. Alternatively, it is possible that only a few founders of tetraploid hybrids are at the origin of all currently observed tetraploid plants and that preferential breeding among plants of this cytotype has provided the opportunity for genetic recombination between the genomes of *R. serbica* and *R. nathaliae*. This would result in a wide variety of genetic combinations from the two parental genomes, including the existence of genetic profiles similar to the parental types (i.e., *R. nathaliae* or *R. serbica*). This latter possibility would imply that synapsis occurs between the chromosomes of the two species, which remains to be proven. However, in that case, one would expect to observe a continuous distribution of ancestry coefficients within the tetraploid group, from “nathaliae” to “serbica” and all possibilities in between. Data reported here showed a different pattern with a clear bimodality in this distribution. The coexistence of divergent genomes in interspecific hybrids is considered as a major mechanism of molecular and phenotypic novelty. This is explained by the huge structural and functional variation caused both by the change in ploidy level, if any, and by novel genetic interaction effects generated between the divergent genomes that merge (see Nieto Feliner et al., 2020, for a review). This variation is a raw material on which selection can operate. For example, negative epistasis can contribute to post-zygotic barriers to interspecific gene flow through a decrease in fitness of interspecific F1 hybrids and/or their progenies (Taylor et al., 2009), limiting therefore genetic introgression into restricted regions of genomes (adaptive introgression, e.g., Marburger et al., 2019). It is therefore possible that, among tetraploid *Ramonda* interspecific hybrids, selection in favor of “parental” combinations of co-adapted genes has taken place, because of weak survival or fertility of individuals having a mixed genome composition. The AFLP patterns of the different classes of each cytotype also strongly suggest that different pathways were involved in the production of individuals of the same ploidy level, the 4*x* first-generation hybrids, but also other more unusual and rare ploidy levels. Three putative triploid plants have been observed that show strong ancestry from *R. nathaliae*. This suggested that these triploids could have been produced by fertilization between an unreduced 2*x* gamete of *R. nathaliae* and either a haploid gamete of *R. nathaliae* or a 1*x* gamete with complete *R. nathaliae* genome complement from an interspecific tetraploid hybrid. The latter case would imply that triploids are restricted to sympatry. In the first case, the presence of some triploid individuals would also be expected in *R. nathaliae* populations, but this has not been reported so far. However, the cytotype variability of monospecific populations of *R. nathaliae* has always been based on only a few individuals, and it cannot be excluded that such triploid individuals exist but have not yet been identified. Indeed, despite the high sample size of the present study, only three potential triploids at sympatry were found, all at Radovanski Kamen. However, it is now known that the production of non-reduced gametes is highly variable between individuals within populations and that this ability is also influenced by environmental conditions (De Storme and Mason, 2014). In addition, mainly because of chromosome pairing during meiosis, triploids are often sterile or at least with greatly reduced fertility (Comai, 2005) and therefore rarely remain in natural populations. The above-described pathway to triploid plants opens another way to explain the existence of tetraploid plants with high ancestry coefficient to “nathaliae.” One could hypothesize at least two explanations. First, it is theoretically possible to obtain such profiles in progeny resulting from fertilization between a reduced gamete of a diploid *R. nathaliae* and a triploid gamete of a triploid *R. nathaliae*. The triploid bridge is considered to be very important for interploid gene flow and polyploidization (Husband, 2004). Secondly, unreduced (2*x*) gametes of *R. nathaliae* may also be involved in the production of tetraploid individuals that possess only chromosomes of *R. nathaliae* by autopolyploidy. The existence of such tetraploid individuals could also open the possibility of obtaining 6*x* progeny with only *R. nathaliae* genomes, the presence of which is suggested by the AFLP data. This hypothesis deserves attention because such tetraploid “nathaliae” would have inherited the ability to produce unreduced gametes (i.e., 4*x* gametes) from their *R. nathaliae* diploid parent.

Therefore, the AFLP profiles of the tetraploid individuals may indicate the existence of an autopolyploid-allopolyploid mixture at both sites of sympatry. They also suggest that there could be some barriers to reproduction between the two tetraploid hypothetical types, since otherwise tetraploid individuals with a much higher genome admixture would arise in the progeny from such crosses, which was not observed. However, the few individuals in our sample that exhibit such an intermediate AFLP profile do not allow us to rule out this last possibility. If this is indeed the case, the tetraploid level could also be a bridge between the two species, reinforcing interploidy gene flow in sympatry.

Pentaploid individuals were also found in both places. They may have originated in different ways. For example, they may simply have come from a cross between a tetraploid (of whatever type) and a hexaploid, or even from the union of an unreduced 2*x* gamete from *R. nathaliae* and a 3*x* gamete from a hexaploid. These pentaploid individuals could also play a role as mediators of gene flow, but without forming an independent evolutionary unit (Tel-Zur et al., 2020; Peskoller et al., 2021).

Obviously, other pathways could be invoked to explain all patterns of GS and AFLP profiles observed in this study. For example, it has been observed (Lazarević et al., 2013) that hybrid individuals produced pollen with highly variable sizes, a well-known proxy for irregular meioses in plants. It is therefore possible that 4*x* plants could produce haploid gametes with one *R. serbica* complement, a pathway for the production of 2*x* progenies with the genetic feature of *R. serbica*, an intriguing pattern we observed in our data.

Further studies are therefore needed to gain a comprehensive and detailed understanding of the biological mechanisms that explain the cytotype and molecular diversity observed at the two sites of sympatry. Further studies should focus on finding evidence for the production of unreduced gametes, especially in *R. nathaliae*, but also in triploids and tetraploids. In particular, a more detailed genetic study of the genetic profiles of the plants found at the two sites of sympatry, based on large-scale sequence data, should be useful to assess the extent of genome mosaicism with respect to each cytotype. This would help to assess the extent of genetic introgression between the two species and the potential for recombination between their genomes. However, it is already clear that hybridization between the two species is occurring and that, at the very least, backcrossing (in which tetraploid hybrids give rise to pentaploids) is possible. Unfortunately, backcrosses with one or both parental species often result in the appearance of individuals whose morphology is difficult to distinguish from the parental species, and identification of hybrids based on morphology becomes very difficult or impossible (Lihová et al., 2007; Čertner et al., 2015; Tendal et al., 2018; Beirinckx et al., 2020), as seems to be the case with both sites of sympatry for *Ramonda* (Lazarević et al., 2014).

Our data (both cytotypes and AFLP markers) therefore suggest that the first-generation interspecific hybrids are viable and possibly fertile. It has already been shown that pollen grains from tetraploid individuals are viable, although somewhat less so than pollen from parental cytotypes in sympatry (Lazarević et al., 2013). On the other hand, seeds of tetraploid individuals are drastically smaller than those of parental species and have very low or no germination capacity (Lazarević et al., 2013), which is often the case in interspecific hybrids (Mallet, 2008; Schmickl and Yant, 2021). Interestingly, with the exception of *R. nathaliae* in Oblik, seeds of both parental cytotypes have shown lower germination rates compared to monospecific populations (Lazarević et al., 2013), which could be the consequence of a reduction in fitness caused by various factors such as genetic introgression or inclusion of pollen from the hybrid individuals in pollination processes (Jacquemyn et al., 2016). The large number of hybrid individuals observed in sympatry suggests that they arise from regular gene flow between parental species in these sites, as shown by Abdelaziz et al. (2021) in two *Erysimum* species. If hybridization events between the two species were rare, competition between the cytotypes would have been expected to eliminate the hybrids due to their rarity (cytotype exclusion), unless there were counteracting factors (homogamy, enlargement of the hybrid niche, or heterosis). Vegetative reproduction may also contribute to their maintenance (Lazarević et al., 2013). It is also possible that the process of minority cytotype exclusion either does not work for some reason that should be investigated or has not yet eroded the diversity of cytotypes in the two sites of sympatry. This last argument could support the idea that the only two known secondary contact zones between the two species are recent and probably formed during repeated glaciations in the Pleistocene. Finally, the overall panorama of genetic and cytotype variability may indicate that a secondary contact zone between the two species emerged after a long period of isolation (Schmickl and Yant, 2021).

## Conclusion

The present study shows the complex influence of various factors on the evolution of two representatives of the genus *Ramonda* in the Balkan Peninsula. The turbulent geological past of the Balkan Peninsula, together with climatic fluctuations throughout the Mediterranean, has influenced the distribution, ecology, speciation and genetic diversity of *Ramonda* species, leading to the formation of isolated populations in refugial habitats. During migrations favored by interglacial periods, a secondary contact zone between *R. nathaliae* and *R. serbica* formed in a narrow area in E Serbia. In this newly formed sympatry, an intense hybridization process takes place between the two species, leading to the occurrence of different ploidy levels (from 2*x* to > 8*x*) and individuals with mixed morphological characteristics, suggesting that not only crosses between the parental species occur, but also backcrosses and possibly duplication of the whole genome. These two sites of sympatry are places for a huge genetic mixing. Moderate pollen fertility, but low seed germination and low dispersal potential suggest that the effects of hybridization are limited to the sites of sympatry. However, the rich mixture of ploidy levels shows that these relict species, which have preserved a specific survival strategy, are not evolutionary “dead ends” and raises the question of possible ecological and evolutionary consequences of this phenomenon. These two locations constitute therefore an open-air laboratory for evolutionary and interspecific recombination studies.

## Data Availability Statement

The original contributions presented in the study are included in the article/Supplementary Material, further inquiries can be directed to the corresponding authors.

## Author Contributions

SS-Y, BS, VS, and TR designed the study. ML, SS-Y, BS, VS, TR, AS, MN, and GT collected plant material. ML and SS-Y performed cytometric measurements and chromosome counts. ML, AS, and FL performed molecular laboratory work and provided data for statistical analyses done by TR and DH. ML, TR, and SS-Y drafted the manuscript. All authors read, revised, and approved the final version of the manuscript.

## Funding

This research was supported by the Ministry of Education, Science and Technological Development of the Republic of Serbia (No. 451–03–68/2022–14/200178), the International Franco-Serbian “Pavle Savić” project, and the Federative Institute of Research (IFR 87 La Plante et son environnement) at Gif-Orsay. The l’Institut Français de Serbie provided grants to ML.

## Conflict of Interest

The authors declare that the research was conducted in the absence of any commercial or financial relationships that could be construed as a potential conflict of interest.

## Publisher’s Note

All claims expressed in this article are solely those of the authors and do not necessarily represent those of their affiliated organizations, or those of the publisher, the editors and the reviewers. Any product that may be evaluated in this article, or claim that may be made by its manufacturer, is not guaranteed or endorsed by the publisher.
