# Supplementary material for: Genetic Variability in Balkan Paleoendemic Resurrection Plants Ramonda serbica and R. nathaliae Across Their Range and in the Zone of Sympatry
Source: Front Plant Sci. 2022 Apr 28;13:873471. doi: 10.3389/fpls.2022.873471 (PMC9096497; doi:10.3389/fpls.2022.873471)

**Supplementary Figure 1.**

Dendrogram built on inter-population DAS genetic distance using *R. serbica* and *R. nathaliae* populations from the large geographical scale study. Reliability of nodes (in %) were computed on the basis of 1000 bootstrap on locus.

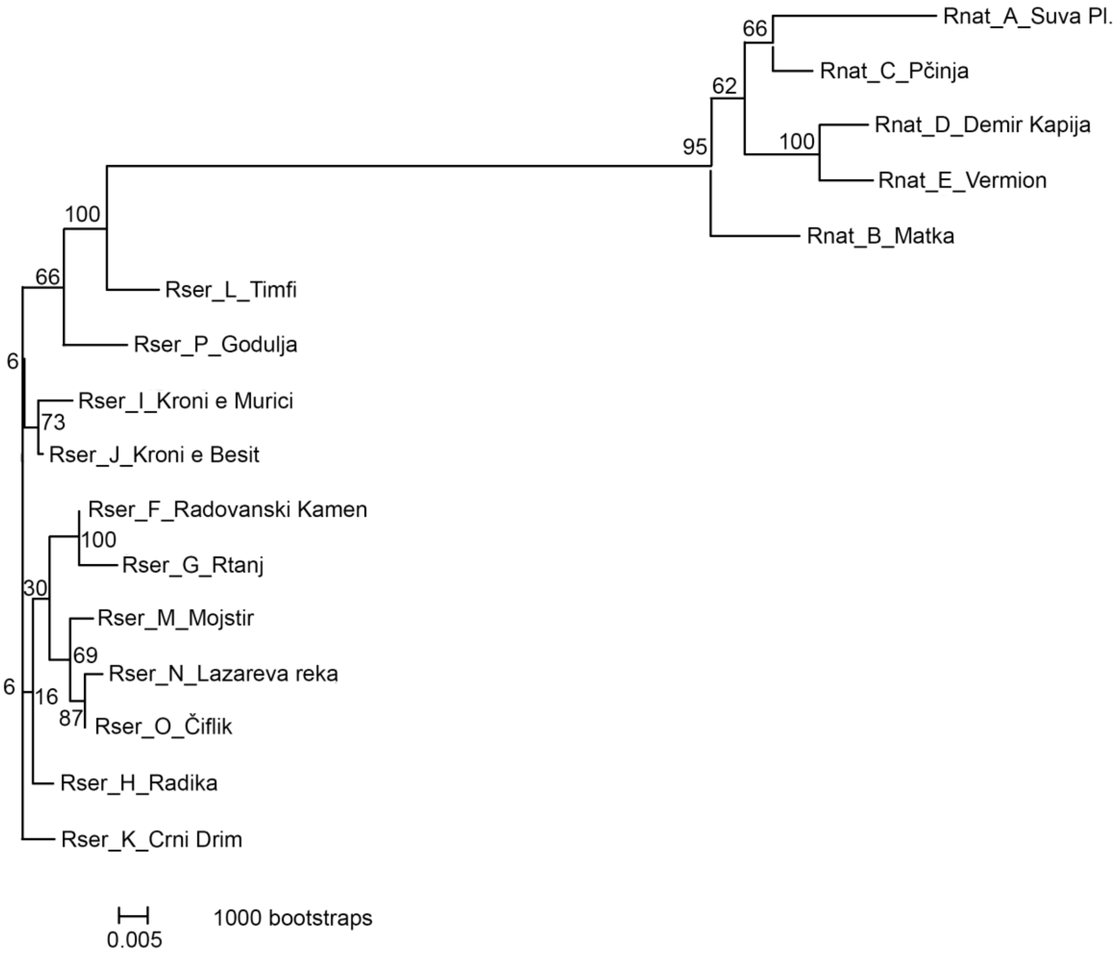

Supplement: Supplementary file 1 [file Data_Sheet_1.PDF]
