# Supplementary material for: Genetic Variability in Balkan Paleoendemic Resurrection Plants Ramonda serbica and R. nathaliae Across Their Range and in the Zone of Sympatry
Source: Front Plant Sci. 2022 Apr 28;13:873471. doi: 10.3389/fpls.2022.873471 (PMC9096497; doi:10.3389/fpls.2022.873471)

Supplementary Figure 2. Histogram of assignments of individuals to monospecific populations of *R. nathaliae* and *R. serbica*. Population codes the same as in Table 1. The assignment posterior probability (expressed in %) is given in brackets for each population.

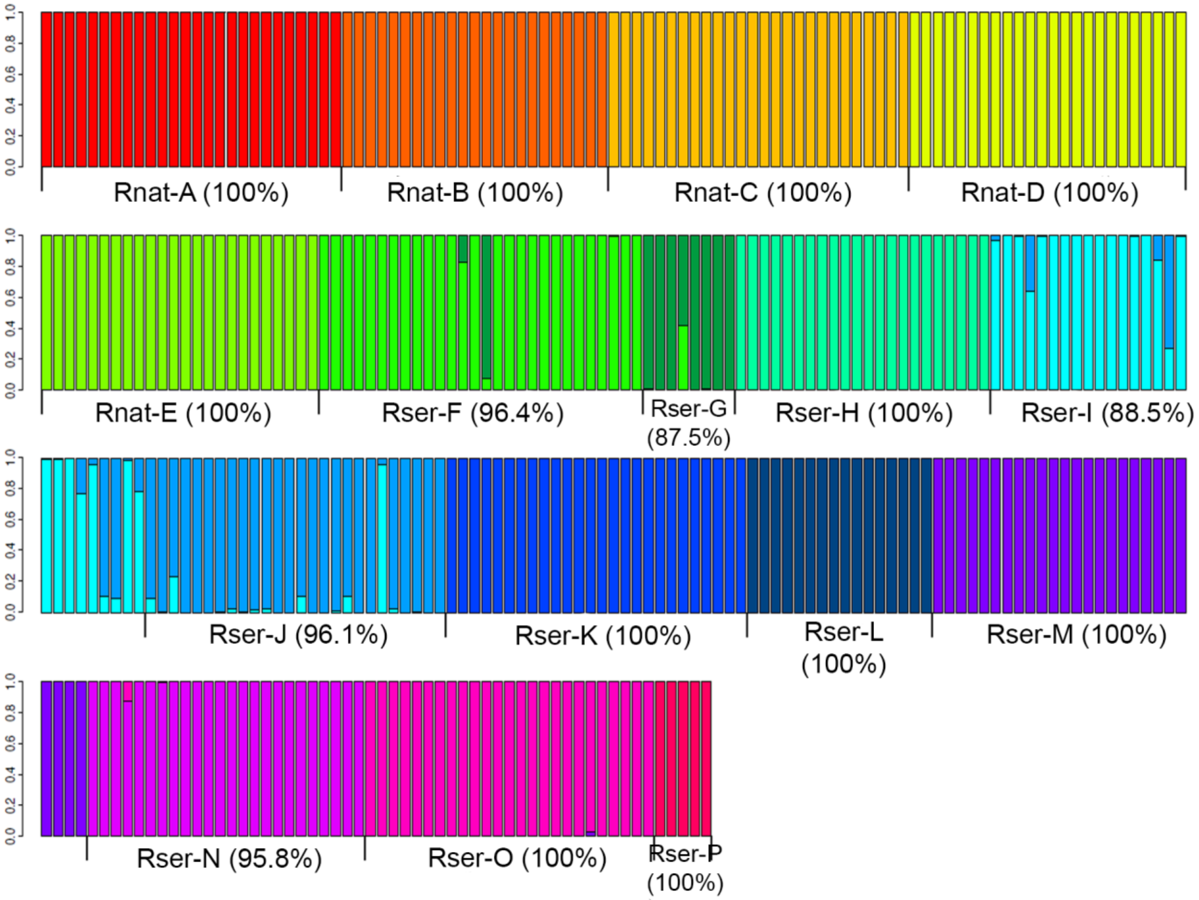

Supplement: Supplementary file 2 [file Data_Sheet_2.PDF]
