# Supplementary material for: Genetic Variability in Balkan Paleoendemic Resurrection Plants Ramonda serbica and R. nathaliae Across Their Range and in the Zone of Sympatry
Source: Front Plant Sci. 2022 Apr 28;13:873471. doi: 10.3389/fpls.2022.873471 (PMC9096497; doi:10.3389/fpls.2022.873471)

Supplementary Figure 3.

Distribution, across loci, of differences in the frequency of the dominant AFLP phenotype (“presence of fragment”) between *R. serbica* and *R. nathaliae*. The phenotype frequencies were computed from samples on monospecific populations used for the study of sympatry.

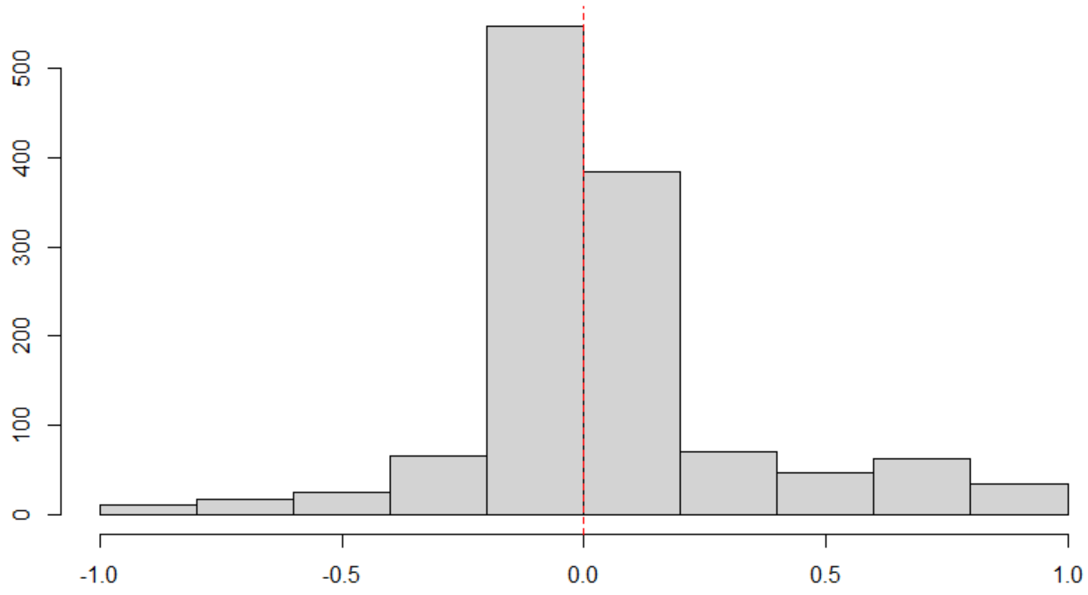

Supplement: Supplementary file 3 [file Data_Sheet_3.pdf]
