# Supplementary material for: Genetic Variability in Balkan Paleoendemic Resurrection Plants Ramonda serbica and R. nathaliae Across Their Range and in the Zone of Sympatry
Source: Front Plant Sci. 2022 Apr 28;13:873471. doi: 10.3389/fpls.2022.873471 (PMC9096497; doi:10.3389/fpls.2022.873471)

Supplementary Figure 4.

Results from the Evanno test showing the best clustering solution was obtained for K=2, according to the ΔK criteria.

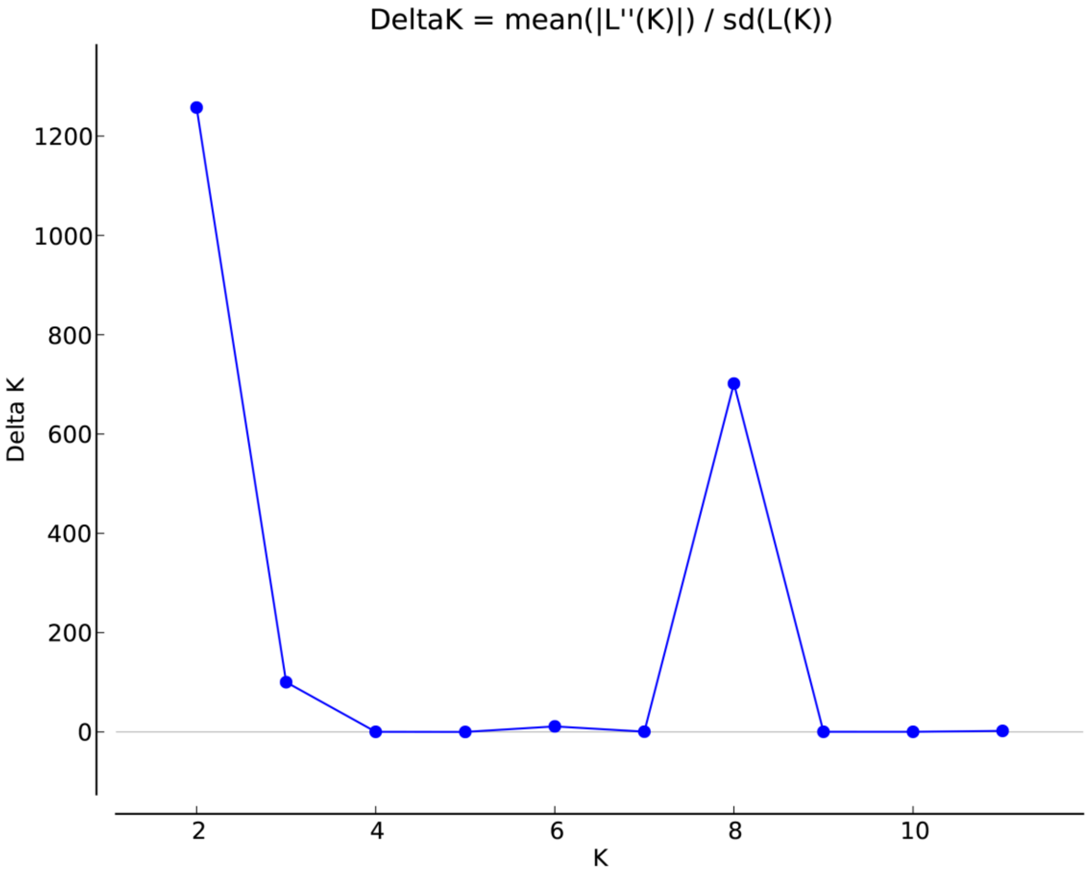

Supplement: Supplementary file 4 [file Data_Sheet_4.PDF]
