# Supplementary material for: Genetic Variability in Balkan Paleoendemic Resurrection Plants Ramonda serbica and R. nathaliae Across Their Range and in the Zone of Sympatry
Source: Front Plant Sci. 2022 Apr 28;13:873471. doi: 10.3389/fpls.2022.873471 (PMC9096497; doi:10.3389/fpls.2022.873471)
Supplement: Supplementary file 5 [file Data_Sheet_5.pdf]

Supplementary Figure 5.

Bar plots displaying ancestry coefficients of individuals from monospecific populations and from the two sites of sympatry, for the best clustering solution (K=2). Individuals are arranged according to their sampling sites. In each sympatry, individuals were further arranged according to their ploidy level. “Other” refer to individuals that could not be undoubtedly assigned to a given ploidy level from their genome size. Each genetic cluster is represented by a specific colour (orange for the “serbica” genetic cluster, and yellow for the “nathaliae” genetic cluster).

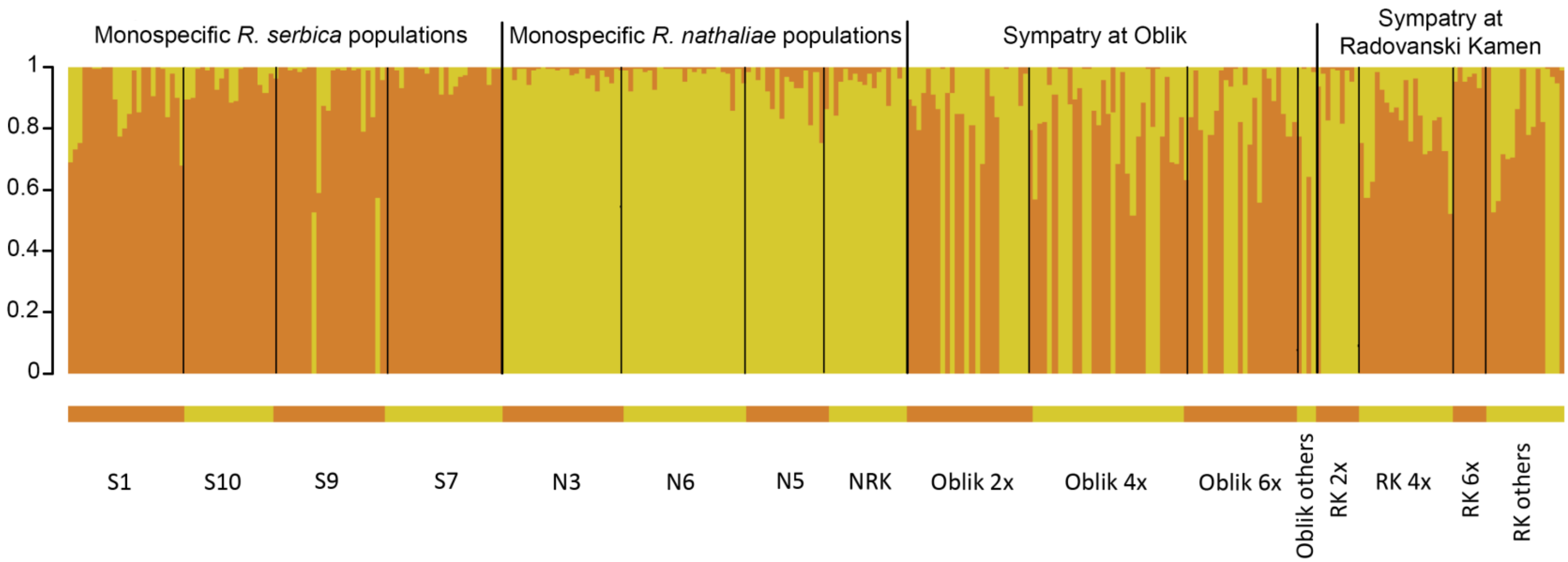

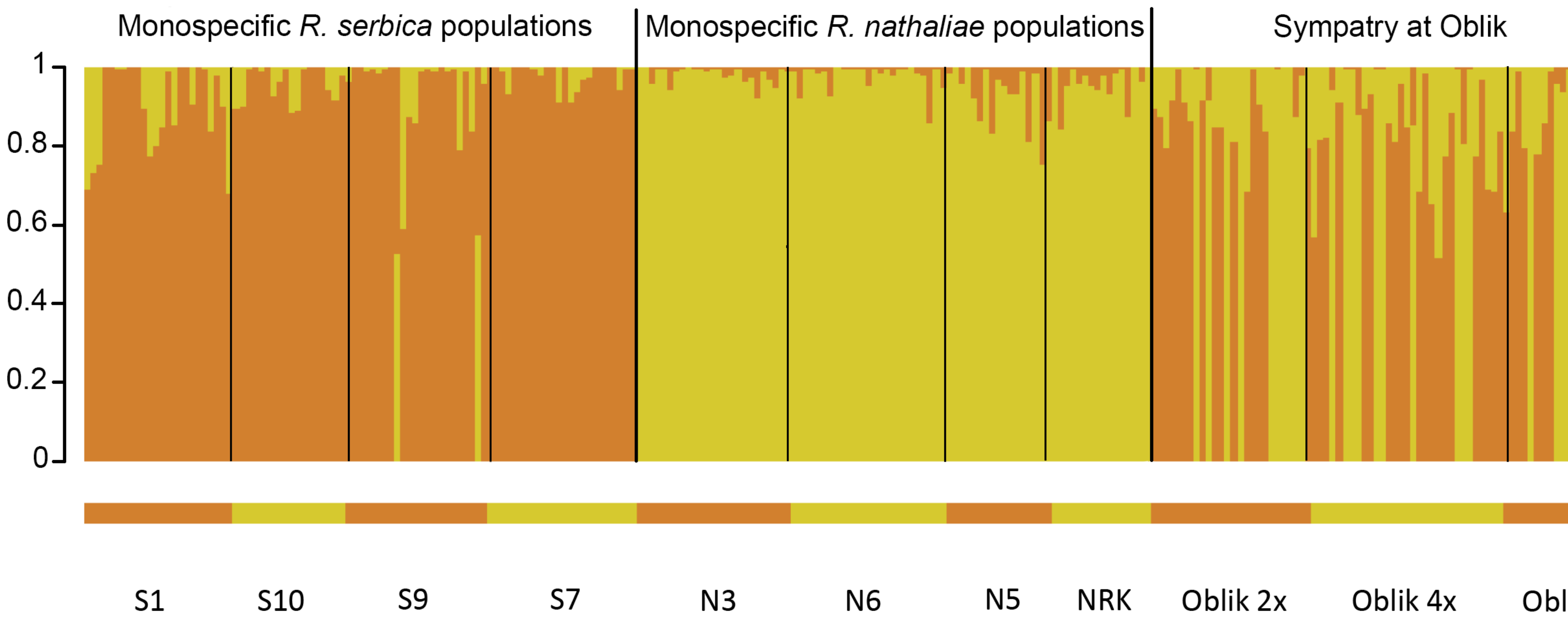

Monospecific R. serbica populations
Monospecific R. nathaliae populations
Sympatry at Oblik
1
0.8
0.6
0.4
0.2
0
S1
S10
S9
S7
N3
N6
N5
NRK
Oblik 2x
Oblik 4x
Obl
