# Supplementary material for: Genetic Variability in Balkan Paleoendemic Resurrection Plants Ramonda serbica and R. nathaliae Across Their Range and in the Zone of Sympatry
Source: Front Plant Sci. 2022 Apr 28;13:873471. doi: 10.3389/fpls.2022.873471 (PMC9096497; doi:10.3389/fpls.2022.873471)

**Supplementary Figure 6.**

Bayesian Criteria Indices (BIC) for DAPC analysis on monospecific populations and populations in sympatry. A clear plate is reached at K=3.

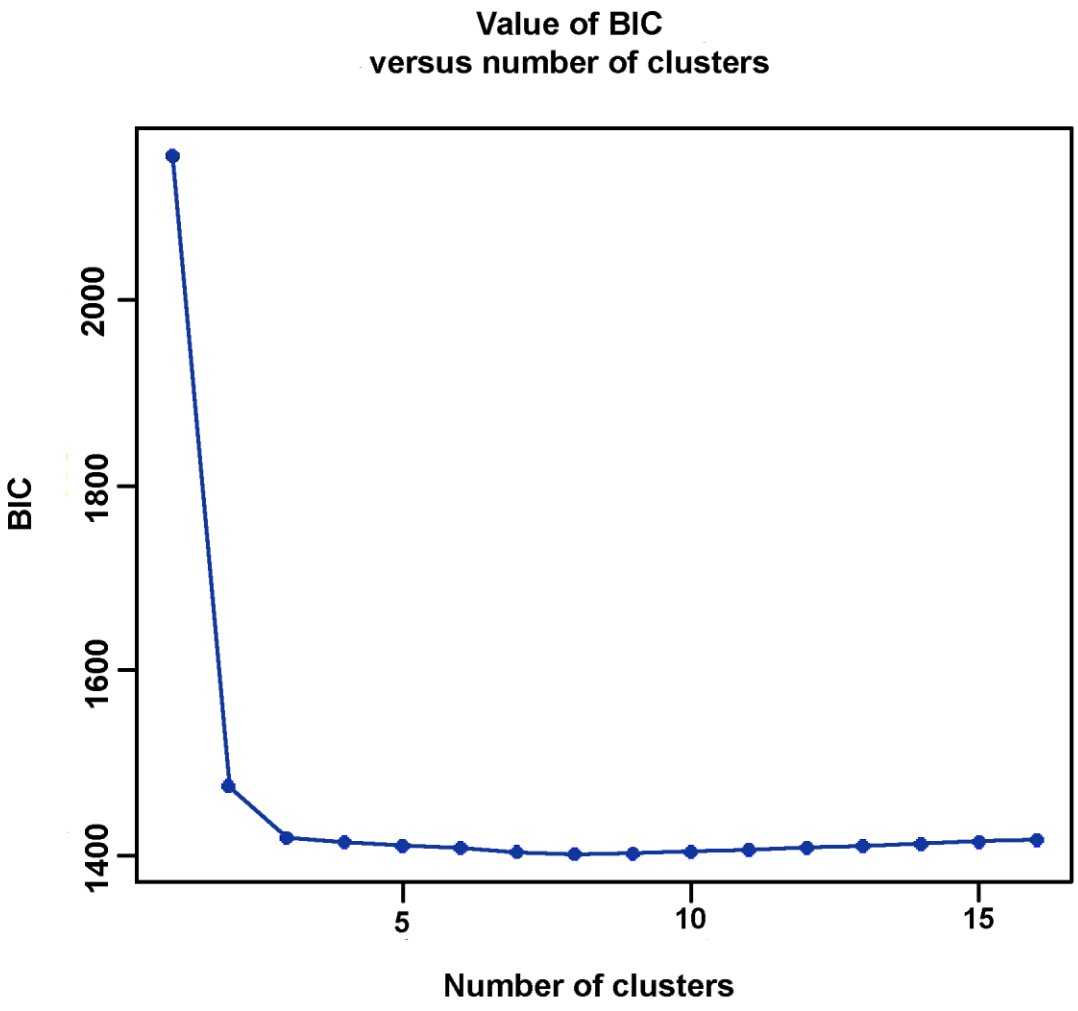

Supplement: Supplementary file 6 [file Data_Sheet_6.pdf]
