# Supplementary material for: Genetic Variability in Balkan Paleoendemic Resurrection Plants Ramonda serbica and R. nathaliae Across Their Range and in the Zone of Sympatry
Source: Front Plant Sci. 2022 Apr 28;13:873471. doi: 10.3389/fpls.2022.873471 (PMC9096497; doi:10.3389/fpls.2022.873471)
Supplement: Supplementary file 7 [file Data_Sheet_7.PDF]

Supplementary Material 1

DNA extraction and AFLP analyses in details

Fragments of silica-gel dried leaves weighing from 21 to 26 mg were used for the DNA extraction performed by NucleoSpin®96 Plant kit (Macherey – Nagel) according to the manufacturer's manual. The quality and quantity of the extracted DNA was checked on a NanoDrop 1000 (Thermo Scientific, Waltham, MA, USA).

AFLP analyses were performed according to the method described by Vos et al. (1995) with some adaptations. Digestions were done in final volume of 25µl including 5U of restriction enzyme EcoRI (10 U/µl), and 5U of Tru1I (10 U/µl), in ligase buffer. After 2 h of incubation at 37°C, enzymes were disabled at 70°C during 20 minutes and products of digestion were then put in the freezer at -20°C. Ligation was done in the total volume of 50 µl containing ligase buffer (10X), T4 DNA ligase (1 Weiss U/µl; MBI Fermentas), and adapters: 5 µM EcoRI (5'-CTCGTAGACTGCGTACC-3'; 3'-CTGACGCATCGTTAA-5'), and 50 µM Tru1I (5'-CAGGATGAGTCCTGA G-3'; 3'-TACTCAGGACTCAT-5'). The incubation was done for 2 h 30 min at 20°C. Ligation products were ten times diluted in sterilized water and put in freezer at -20°C. Samples for two studies were digested and ligated separately, but all samples for one study were processed during the same manipulation.

Pre-amplification was done by primers complementary to adapters with one additional basis at 3' end. PCRs were done in 50 µl volume containing: 5 µl template DNA (digested and ligated) ten times diluted, 5 µl of buffer (10X) without $MgCl_2$, 5 µl $MgCl_2$ (25 mM), 4 µl dNTPs (2.5 mM each), 1 µl of 10 µM EcoRI primer (5'-ACTGCGTACCAATTC-3'), 1 µl of 10 µM Tru1I primer (5'-GATGAGTCCTGAGTAA-3') and 1 µl Taq polymerase (5U/ µl; MP Biomedicals, QBiogene, France). PCR follows the program in three steps: denaturation at 94°C for 30 sec, followed by annealing at 56°C for 1 min and elongation at 72°C for 1 min, all this for 20 cycles. To verify the quality of amplifications, 9 µl of several randomly selected pre-amplification products were tested by migration on 2 % agarose gel for 1 h at 100 V. Pre-amplifications were then 10 times diluted in sterilized water and put in freezer at -20°C.

Three primer combinations were selected for full analysis (fluorescent dyes in parentheses): in both studies EcoRI-AAC/MseI-CTC (FAM) and EcoRI-CAG/MseI-CTC (VIC) were used, while the third primer pair differed EcoRI-ACA/MseI-CTC (NED) being used in the first AFLP experiment (monospecific populations only), and EcoRI-CAG/MseI-CTT (VIC) in the second experiment (hybridization study).

Amplifications were performed in 25 µl volume containing 4 µl of pre-amplification product diluted 10 times in sterilized water, 2.5 µl of buffer (10X) without $MgCl_2$, 2.5 µl $MgCl_2$ (25 mM), 2 µl dNTPs (2.5 mM each), 0.5 µl of 10 µM fluorescent primers EcoRI, 0.5 µl of 10 µM primers Msei and 0.2 µl Taq polymerase (5 U/µl; MP Biomedicals, QBiogene, France). PCR program begins with a "touch down" from 65 to 56°C, then: denaturation at 94°C for 1 min, annealing at 65°C (1°C lower in each cycle) for 1 min and elongation at 72°C for 1 min 30 sec, all this for 10 cycles. Program finishes by 23 cycles that included three steps: denaturation at 94°C for 30 sec, annealing at 56°C for 30 sec and elongation at 72°C for 1 min. Products of these amplifications were further kept at -20°C.

Samples were prepared for fragment detection on the capillary sequencer by diluting 3 µl of amplification product in 190 µl of water. Then, 3 µl of this dilution were added to 5 µl of formamide with molecular weight marker GeneScan 500LIZ® standard size (Applied Biosystems). The plates are then denaturated for 3 minutes at 95°C and directly put on ice before deposited on sequencer.
