# Supplementary material for: Genetic Variability in Balkan Paleoendemic Resurrection Plants Ramonda serbica and R. nathaliae Across Their Range and in the Zone of Sympatry
Source: Front Plant Sci. 2022 Apr 28;13:873471. doi: 10.3389/fpls.2022.873471 (PMC9096497; doi:10.3389/fpls.2022.873471)
Supplement: Supplementary file 8 [file Data_Sheet_8.PDF]

Supplementary Table 1. Proportion of polymorphic loci (PLP, %) and expected heterozygosity (Hj) obtained by Bayesian method with non-uniform prior distribution. n: sample size; #loc P: number of polymorphic loci per population; S.E.(Hj): standard error for the expected heterozygosity, per population.

**Pop ID**	**Population name**	**n**	**#loc P**	**PLP**	**Hj**	**S.E.(Hj)**
popA	Rnat_Suva pl	26	244	20.6	0.062	0.00380
popB	Rnat_Matka	23	419	35.4	0.128	0.00462
popC	Rnat_Pcinja	26	301	25.5	0.085	0.00433
popD	Rnat_Demir Kapija	24	339	28.7	0.101	0.00435
popE	Rnat_Vermion	24	305	25.8	0.088	0.00440
popF	Rser_Rad. Kamen	28	361	30.5	0.100	0.00455
popG	Rser_Rtanj	8	332	28.1	0.087	0.00442
popH	Rser_Radika	22	381	32.2	0.107	0.00474
popI	Rser_Kroni e Murici	26	348	29.4	0.092	0.00445
popJ	Rser_Kroni e Besit	26	358	30.3	0.109	0.00481
popK	Rser_Crni Drim	26	358	30.3	0.109	0.00474
popL	Rser_Timfi	16	454	38.4	0.140	0.00509
popM	Rser_Mojstir	26	341	28.8	0.087	0.00432
popN	Rser_Lazareva reka	24	375	31.7	0.121	0.00506
popO	Rser_Ciflik	25	343	29	0.095	0.00453
popP	Rser_Godulja	5	326	27.6	0.105	0.00485
