# Supplementary material for: Genetic Variability in Balkan Paleoendemic Resurrection Plants Ramonda serbica and R. nathaliae Across Their Range and in the Zone of Sympatry
Source: Front Plant Sci. 2022 Apr 28;13:873471. doi: 10.3389/fpls.2022.873471 (PMC9096497; doi:10.3389/fpls.2022.873471)
Supplement: Supplementary file 9 [file Data_Sheet_9.PDF]

Supplementary Table 2. AMOVA results performed for two species together, as well as for every species separately.

	R. nathaliae* + *R. serbica	***R. nathaliae***	***R. serbica***
**Source of variation**	Variation (%)		
**Among groups**	48.51		
**Among populations within groups**	9.91	27.83	13.8
**Within populations**	41.58	72.17	86.2
***Φ*sc**	0.193		
***Φ*st**	0.584	0.278	0.138
***Φ*ct**	0.485		
