# Supplementary material for: Genetic Variability in Balkan Paleoendemic Resurrection Plants Ramonda serbica and R. nathaliae Across Their Range and in the Zone of Sympatry
Source: Front Plant Sci. 2022 Apr 28;13:873471. doi: 10.3389/fpls.2022.873471 (PMC9096497; doi:10.3389/fpls.2022.873471)
Supplement: Supplementary file 10 [file Data_Sheet_10.pdf]

Supplementary Table 3. Classification of individuals on the basis of posterior probabilities computed in the DAPC analysis.

Ecology	Population/Ploidy	Cluster 1	Cluster 2	Cluster 3
**Monospecific populations**	**S1**	24	0	0
	**S10**	18	0	0
	**S9**	22	1	0
	**S7**	23	0	1
	**N3**	0	25	0
	**N6**	0	25	0
	**N5**	0	17	0
	**Nrk**	0	16	0
**Sympatry**	**Oblik_6*x***	18	5	0
	**Oblik_2*x***	16	10	0
	**Oblik_4*x***	21	10	0
	**RK_6*x***	7	0	0
	**RK_2*x***	1	8	0
	**RK_4*x***	19	0	0
	**Oblik_others**	2	2	0
	**RK_others**	15	4	0
